# Biomaterials for Three-Dimensional Cell Culture: From Applications in Oncology to Nanotechnology

**DOI:** 10.3390/nano11020481

**Published:** 2021-02-13

**Authors:** Tarek Saydé, Omar El Hamoui, Bruno Alies, Karen Gaudin, Gaëtane Lespes, Serge Battu

**Affiliations:** 1 EA3842-CAPTuR, GEIST, Faculté de Médecine, Université de Limoges, 2 rue du Dr Marcland, 87025 Limoges, France; tarek.sayde@unilim.fr; 2 ARNA, INSERM U1212, UMR CNRS 5320, Université de Bordeaux, 146 rue Léo Saignat, 33076 Bordeaux, France; elhamoui.omar@gmail.com (O.E.H.); bruno.alies@u-bordeaux.fr (B.A.); karen.gaudin@u-bordeaux.fr (K.G.); 3 CNRS, Institut des Sciences Analytiques et de Physico-Chimie pour l’Environnement et les Matériaux (IPREM), UMR 5254, Université de Pau et des Pays de l’Adour (E2S/UPPA), 2 Avenue Pierre Angot, 64053 Pau, France

**Keywords:** 3D cell culture, oncology, nanoparticles, nano-toxicity

## Abstract

Three-dimensional cell culture has revolutionized cellular biology research and opened the door to novel discoveries in terms of cellular behavior and response to microenvironment stimuli. Different types of 3D culture exist today, including hydrogel scaffold-based models, which possess a complex structure mimicking the extracellular matrix. These hydrogels can be made of polymers (natural or synthetic) or low-molecular weight gelators that, via the supramolecular assembly of molecules, allow the production of a reproducible hydrogel with tunable mechanical properties. When cancer cells are grown in this type of hydrogel, they develop into multicellular tumor spheroids (MCTS). Three-dimensional (3D) cancer culture combined with a complex microenvironment that consists of a platform to study tumor development and also to assess the toxicity of physico-chemical entities such as ions, molecules or particles. With the emergence of nanoparticles of different origins and natures, implementing a reproducible in vitro model that consists of a bio-indicator for nano-toxicity assays is inevitable. However, the maneuver process of such a bio-indicator requires the implementation of a repeatable system that undergoes an exhaustive follow-up. Hence, the biggest challenge in this matter is the reproducibility of the MCTS and the associated full-scale characterization of this system’s components.

## 1. Introduction 

The closer we can get to mimicking the human condition, the better the scientific research is going to be toward understanding the fundamental pathology of a disease, and also toward predicting patient response regarding drug therapy and estimating the toxicity of certain physico-chemical entities surrounding us on a daily basis. Biomaterials will allow us to recreate a smaller version of our inner physiological microenvironment, making it possible to host cells and allow the diffusion of small entities such as nanomaterials.

Cell culture is an indispensable tool for understanding the fundamental biophysical and bio-molecular mechanisms by which cells assemble into tissues and organs; and to understand the physiological functions of cells and consequently their disruption during illness. Nowadays, cell culture is used in biomedical research, tissue engineering, regenerative medicine as well as other industrial practices.

Accordingly, these in vitro cell cultures serve as a platform to understand the in vivo cellular behavior such as migration and differentiation. Nevertheless, conventional two-dimensional (2D) culture systems may give results that deviate from the true in vivo response. To overcome this limitation, new three-dimensional (3D) cell culture platforms are designed to better mimic in vivo conditions [1]. Cell lines provide us with excellent materials for biological studies and 3D culture leads these cells to behave in a way close to the natural conditions existing in the organism [2].

**Figure 1:**
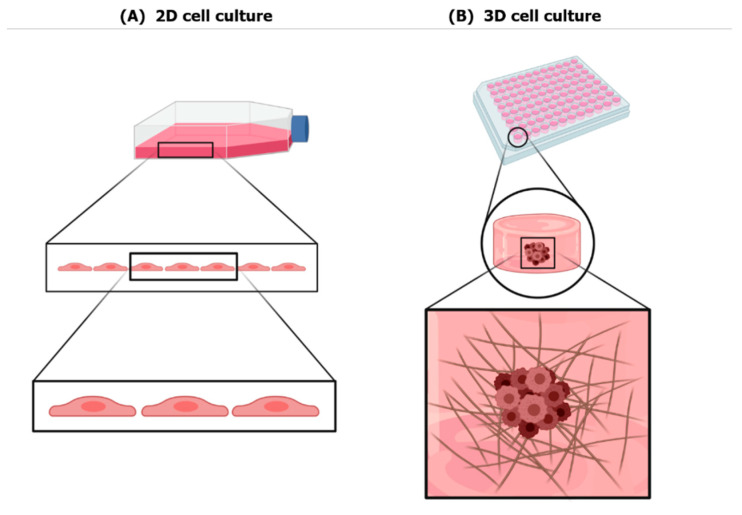
2D vs. 3D cell culture: (**A**) Cell behavior in 2D conventional cell culture. Cells cultured in a 2D manner tend to have a flat shape that does not represent the real physiological cell morphology. (**B**) Cell behavior in 3D cell culture. Cells cultured in a 3D system are present in a microenvironment similar to that in vivo, therefore they have a more representative morphology and behavior

Under conventional 2D culture conditions, components of the extracellular matrix (ECM) as well as cell–cell and cell–ECM interactions, which are important for differentiation, proliferation and normal cell function in vivo, are altered [3]. In addition, 2D culture methods do not provide control over cell shape, which is a biophysical parameter affecting cell bioactivity in vivo [4]. Cells in the body have bioactivity stimuli dependent on their 3D microenvironment as cell–cell and cell–ECM interactions affect basic cellular behavior and even organ function. Historically, previous studies have shown that 3D cell organization reveals unanticipated results on the mechanism of tumorigenesis. These discoveries suggest that 2D in vitro cancer studies display shortcomings. 3D cell culture increases the dimensionality of the ECM around the cells. Thus, the major advantage of 3D culture over 2D culture is the reduction of the gap of cell behavior between in vitro cell culture systems and in vivo cell physiology [4,5,6] (Figure 1).

In order to confirm an observation made in vitro, the most reliable approach and most common is to use standard animal models such as mice, zebra fish, etc. [7].

However, there are numerous worries rising today concerning in particular the amount of pain and discomfort that an animal model has to endure. A fair amount of the animals that go through testing have a jeopardized immune system and do not possess a tumor–microenvironment interaction similar to that of humans. Consequently, it weakens the reliability of the given results. This prevents an efficient translation of novel research to clinical settings [8]. In addition, experiments including animal trials can be time consuming and very expensive. The 3D models are a credible alternative to the use of animals and meet the ethical rule of the 3Rs (Reduce, Replace, Refine) limiting the use of animal experimentation [2,9,10,11,12].

A continuous challenge that we face today is obtaining a conformity between animal testing and clinical trials [13]. Therefore, the need to create cellular models that can better capture the complexities of research in biology is a motivation to switch from 2D to 3D culture [14]. By replacing in vivo models with 3D cultures, the latter forms a bridge between 2D culture and animal testing.

3D models have proven their potential in many applications in today’s research that we will be discussing later on. Nevertheless, the core of this review is to explain their role in nanotechnology, in particular how these 3D cellular models can serve as a platform to study nano-toxicity, especially their impact on living systems including human organisms. This field has been insufficiently exploited and demands urgent attention due to the complexity in sample preparations as well as in vitro models implementation [15]. While presenting a panel of 3D systems alongside their advantages and inconveniences, we will be mainly focusing on hydrogels that have particular characteristics and their implications in nano-toxicity assays.

## 2. Three-Dimensional Culture Systems

In the preclinical development and translation of nanotechnology-based oncology platforms, cell-based biological systems that closely mimic tumors are vital. [16] A single 3D technology, consisting as a solution or remedy to all difficulties or diseases, does not exist. Numerous approaches have been developed to meet the expanding interest in 3D cell culture. Therefore, scientists are required to select the most suitable model for their cell-based assay [1]. A 3D system must answer to key characteristics such as retaining the natural shape of the cell [17], allowing heterogeneous exposure between the cell interface and medium as in the physiological state there is a gradient availability of media components [18]. Cell junctions should be prevalent and enable cell-to-cell communication [19]. Particularly, a 3D system should permit a high cell viability as observed in vivo [20], and cell proliferation [21].

Two broad categorizations for 3D culture exist: culture systems scaffold-free or scaffold-based, with the latter being either natural or synthetic.

### 2.1. Scaffold-Free Systems 

**Figure 2:**
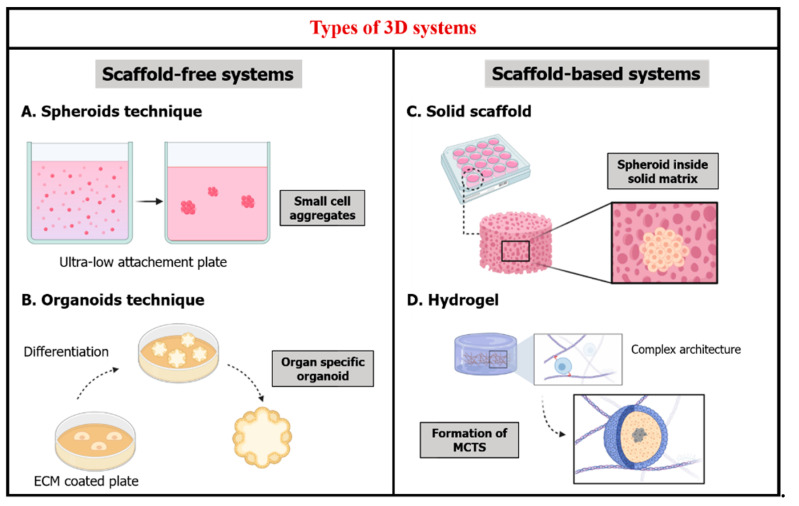
Types of 3D systems. 3D systems fall into two categories either scaffold-free or scaffold-based systems. Scaffold-free systems depend greatly on the plate where the cells are culture whether it is (**A**) ultra-low attachment plate for the production of cell aggregates as spheroids or (**B**) Extracellular matrix (ECM) coated plates for cell differentiation into organoids. Scaffold-based systems are manmade microenvironments that can host cells whether they are (**C**) solid scaffolds that offer a rigid matrix and allow spheroid formation or (**D**) soft scaffolds like hydrogels that contain an ECM-like complex architecture in which multicellular tumor spheroids (MCTS) are produced similar to solid in vivo tumors.

The *spheroids technique* consists of a model with different layers of cells that compensates the deficiencies seen in monolayer cultures [1,22]. This model can be obtained in different methods such as the use of low-adhesion plates to promote the self-aggregation of cells into spheroids [16,23] (Figure 2A), the use of hanging drop plates [9] or the use of bioreactors to produce such models under dynamic culture conditions [24].

However, spheroid culture is accompanied by several limitations that include the difficulty in production of spheroids having a uniform size and standardized composition, out of a small number of seeded cells; and an absence of adequate standardized assays regarding drug-screening studies therefore narrowing the spectrum of applications of these models [22].

The *organoids technique*, that are “a collection of organ-specific cell types that develop from stem cells or organ progenitors and self-organize through cell sorting and spatially restricted lineage commitment in a manner similar to in vivo” [25]. It either is obtained by a monolayer culture of cells on feeder cells or on a surface coated with an ECM, for example fibroblasts, so by cell differentiation, organoids are formed (Figure 2B) [22]. Alternatively, there is a second approach by differentiating primary tissues [26].

Studies have shown that, even though this model present an in vivo-like architecture and complexity, its main limitations reside in its variability and amenability to withstand high-throughput screening (HTS) and high-content screening (HCS) [27].

### 2.2. Scaffold-Based Systems

Another more important and complex facet of 3D culture is scaffold-based systems. This is a discipline that extents the range of options available to scientists. 

Scaffolds are described as fabricated 3D structures made of an array of materials having different porosities, permeability, surface nature and mechanical stability that are modulated in order to design an architecture representing an ideal reconstruction of the microenvironment of specific tissues, also known as the ECM.

Unlike scaffold-free systems, this micro architecture enhances the biophysical and biochemical interaction of the adhered cells and provides a biologically active matrix for the cells to proliferate, differentiate and auto-organize. 

Two subcategories emerge from this discipline:

- *Solid scaffold-based technology* that provides a 3D space hosting cells and allowing them to create 3D tissue-like structures. Natural or synthetic, they consist of porous membranes or fibrous scaffolds that have been widely studied in the field of stem cells and regenerative medicine, for example, porous membranes produced by thermally induced phase separation [28,29]. Owing to their porous structure, these 3D matrixes facilitate tissue regeneration (e.g., cornea, skin and bone) [30,31,32]. Other types of solid scaffold-based technologies are 3D tissue models made of paper-based microfluidics. The latter are materials retrieved from plant tissue in the perspectives of developing human tissue structures compatible for 3D culture of mammalian cells [33]. Nanocellulose-based scaffolds, in particular nanocrystalline cellulose and nanofibrillated cellulose [34], and silk-based composite scaffolds [35] have proven their potential in regenerative medicine such as wound healing and organ reparation, due to their special permeability and hemocompatibility.

The interconnected network implemented in the inner matrix present a 3D microenvironment assisting the progress of different indispensable cellular activities like migration, proliferation and cellular interactions [36] (Figure 2C).

Nonetheless, this scaffold-based system demands laborious synthesis and construction steps. It is equally challenging to seed and incorporate cells inside this matrix [37]. The cells tend to exhibit inhomogeneous distribution within the microenvironment hence giving unstandardized results. This behavior is widely observed in large constructs with low seeding density [38].

For this matter, a biodegradable, biocompatible and tunable scaffold-based system that is able to host cells in a homogeneous manner is mandatory.

With the advent of technology, the design of a scaffold that meets the requirement of a reproducible 3D culture was brought to life.

- *Hydrogels* that can be designed as a soft scaffold for hosting cells.

Hydrogels are matrixes made of molecules that have the ability to swell but without dissolving in water. This class of materials has a high affinity to its solvent, which explains its swelling properties [39].

Today, hydrogels attract material scientists and biomedical researchers, to which their formulations and applications undergo until this day a series of improvements and optimizations. In their entirety, hydrogels are water based, 3D viscoelastic networks, which allow the diffusion of entities throughout their nano-pores or interfibrillar spaces, and attachment of cells [40].

Hydrogel materials exhibit characteristics of biocompatibility and high permeability for nutrients, oxygen and other water-soluble metabolites, rendering them ideal candidates as scaffolds for cell encapsulation [41].

Hydrogel networks can contain both permanent junctions and semi-permanent junctions like chain entanglements. These architecture’s properties, such as stiffness, swelling features and molecules transportation, are highly affected by the cross-linkage type and branching degree [42]. The most common are either polymeric gels, formed by bioinspired-designed units linked by covalent bond, or low-molecular-weight gelators (LMWG) often mentioned as supramolecular gels since they consist of units self-assembled by weak interactions [43].

Hydrogels have their own properties and natures, yet they exhibit some similarities that could give interest for cell culture. Several of them tend to mimic the ECM architecture: cells encapsulated in a fibrillary environment, formed by a network of covalently-linked or self-assembled units, where they can recover their spatial organization and three-dimensional specificities such as cell–cell and cell–ECM interactions [44].

Further, these hydrogels mimic the ECM architecture in a morphological and biophysical manner. Most of the hydrogels designed to host cells inside of them have rheological properties (i.e., stiffness), often close to living tissue, leading to a cellular development and behavior close to that in vivo without the intervention of animal models [45].

Hydrogels hold many advantages over other types of cell encapsulating systems. They resemble natural soft tissue by comparison to other types of polymeric biomaterials due to the fact that hydrogels are water-swollen entities [39,40,41,42,43,44,45]. They also have advantages over other types of polymeric scaffolds, such as easy control of structural parameters ensuring biocompatibility and a scaffold having adjustable architecture [45]. Hydrogels also provide a complex three-dimensional tissue architecture and complex interactions between ECM and cells [46]. On the biological analysis level, unlike scaffold-free methods, hydrogels are applicable to microplates, amenable to HTS/HCS, are highly reproducible and offer a platform for co-culture assays [22]. 

Hydrogel design is solely based on the nature of their constituent units [47]. Depending on the desired experiments’ end-result, researchers can design a hydrogel that fits their needs. Materials for hydrogel design can either be natural polymers, synthetic polymers or low-molecular-weight gelators.

Natural polymers are used to create natural hydrogels, and different groups can be found:

- Proteins used historically in stem cell-based tissue engineering [2,9,22,45] such as collagen used for example in differentiation of human embryonic stem cells into hepatocytes [48,49], gelatin proved efficient in chondrogenic differentiation of adipose-derived adult stem cells [50,51], fibrin for differentiation of murine embryonic stem cells into neural lineage cells [52,53], and silk [54,55].
- Polysaccharides that are naturally found in the ECM [9,45] such as hyaluronic acid that is either used as an integral scaffold for tissue engineering [56,57] or used as a functionalization tool of synthetic biomaterials to create a hybrid more biomimetic matrix [58] and chitosan for tissue engineering applications [59].
- Matrigels, that originated from murine sarcomas ECM, are basement membrane matrixes composed mainly of four ECM proteins: laminin (60%), collagen IV (30%), entactin (8%) and heparin sulfate proteoglycan perlecan (2%) [60]. By virtue of their built-in bioactivity, they exhibited diverse applications in cellular biology [61]. They have been used in cancer invasion studies to assess the metastasis capabilities of cancer cells [62]. Matrigels were used in cell culture of human pluripotent stem cells (hPSCs) to understand the impact of the micro-environmental factors on cell differentiation [63], for cardiomyocytes as in vitro models of heart activity [64] and organoid production [65]. However, matrigels construction requires a large number of structural proteins but also involves other factors such as growth factors [66] and transcription factors [67]. Previous studies displayed major inconvenience that can limit cellular behavior studies linked to the matrigel’s complex, unclear and highly fluctuating composition [66,67,68].

Hydrogels made of natural polymers exhibit very important advantages much needed in biomedical applications that are biocompatibility, biodegradability and nontoxicity [47]. In addition, they are expected to have better interaction with cells enhancing the occurrence of cell proliferation and differentiation [69].

However, for the purposes of cell culture, high uncontrollable biodegradation capacities of the scaffold can end up being an inconvenience more than an advantage because it establishes a variable that is hard to control and may influence cell activity in unknown ways [9]. Other limitations may include batch-to-batch variabilities in biochemical and mechanical properties. For instance using atomic force microscopy, Soofi et al. showed that a batch of matrigel exhibited featured an average elastic modulus also known as stiffness of 400 Pa whereas another batch displayed an elastic modulus of 840 Pa, twice as high [70]. Other variabilities are observed in soluble factors like peptide and protein concentration [66,68,71]. These variabilities hinder the reproducibility of the results. They also have a higher probability of xenogenic contamination in the likes of viral contaminants [72].

To overcome these limitations, researchers resorted to non-animal synthetic hydrogels made of synthetic polymers that are more reproducible in terms of biochemical and mechanical properties.

Synthetic polymers are also found in different groups:

- Biodegradable biomaterials including poly(lactic acid) (PLA), poly(glycolic acid) (PGA), poly(vinyl alcohol) (PVA) and their copolymers. They are commonly used in regenerative medicine for differentiation assays of stem cells progenitors [73,74].
- Non-biodegradable biomaterials such as poly(ethylene glycol) (PEG) [75], 2-hydroxyethyl methacrylate (HEMA) and acrylamide (AAm) [76]. They maintain physical and mechanical integrity for non-biodegradable applications in tissue engineering [45]. Today non-biodegradable biomaterials are more used combined with biodegradable materials to help tune and control some of their properties such as porosity and permeability.

Synthetic polymers possess tunable mechanical strength because of the strong covalent bonds within their matrix [40,47]. These characteristics are known to affect cell differentiation [77] and cell adhesion [78] so the preference of the desired synthetic polymer depends on the anticipated cellular behavior.

A synthetic scaffold provides reproducibility [9] by minimizing the inter batches quality fluctuation since they are often processed and manufactured, hence there will be more consistency between cultures. These hydrogels possess high water content, enabling transport of oxygen, nutrients, waste, and soluble factors, all of which are important to cell functions [79]. However, several studies have shown that this theory does not apply on all these polymers. They suffer often from several limitations including poor biocompatibility, toxicity, pro-inflammatory unwanted activity etc. [80].

Low-molecular-weight gelators (LMWG) are an alternative for synthetic polymers. They are small-molecule-based hydrogels, and are emerging as a promising tool [80]. The common definition of LMWG includes two key features: (1) they consist of molecules with a molecular weight of at most 3000 Da, (2) they are capable of self-assembling by non-covalent interactions such as hydrogen bonds, electrostatic and Van der Waals interaction, π-stacking or other low energy interactions. 

**Figure 3:**
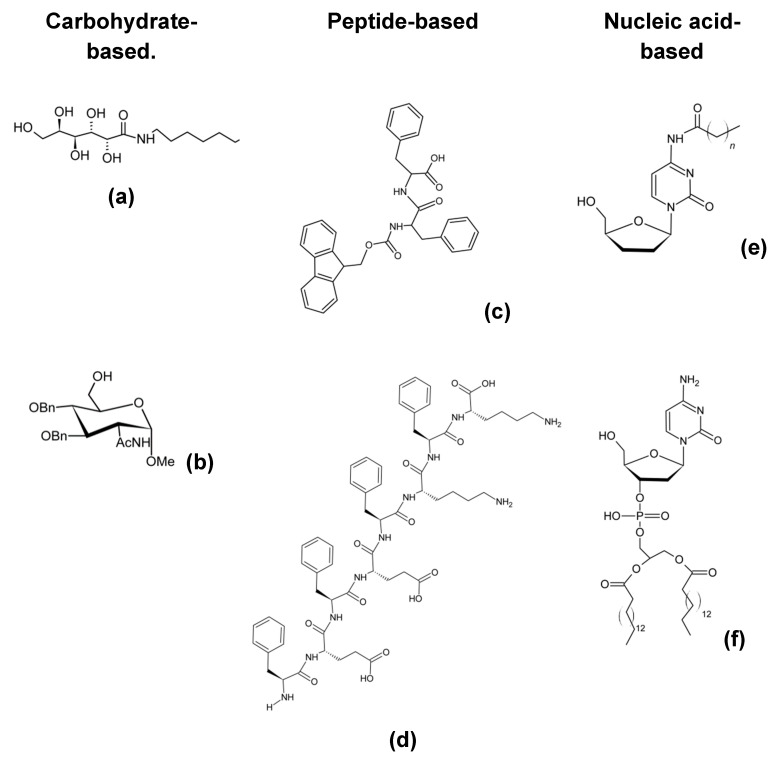
Examples of low-molecular-weight gelators (LMWG) structures. LMWG are either carbohydrate-based, such as (**a**) N-heptylgalactonamide or (**b**) N-acetyl glucosamine, or they can be peptide-based, such as (**c**) FmocFF or (**d**) FEFEFKFK peptide. Alternatively, they can be nucleic acid-based such as (**e**) N-acyl cytidine derivative or (**f**) Nucleotide lipid (diC16dC).

Nowadays, we know several different bio-inspired designs of LMWG, of which the most spread are: (Figure 3)

- Carbohydrate [81,82,83,84]
- Peptide [85,86,87,88,89]
- Nucleic acid based [90,91,92,93].

They have excellent biocompatibility and biodegradability, modular structure that permits specific interactions and most importantly they form a nano-fibrous network that mimics the natural ECM fibrillary architecture [94,95,96].

Carbohydrate-based hydro-gelators are less used in life science than peptide-based, although some of them have shown their successful application as cell culture scaffold, owing to their non-cytotoxicity, their very simple structure and their easy synthesis [83,84].

Amphiphilic-peptides (PAs) are found amidst the self-assembling peptides family. The amphiphilic nature of the molecules constitutes the most relevant feature of PAs.

This feature is a key structure allowing the formation of nanofibers by PAs self-assembly, followed by intertwining to form a hydrogel network [89].

Studies conducted on these molecules showed that the nano-fibrous network resulting from these self-assembling peptides have the ability to form stable hydrogels for cell encapsulation [45,97]. Some other design strategies showed their success by creating bio-inspired derivatives. For example, nucleolipids are perfect molecular design candidates for this type of target hydrogels, because they are made of lipids covalently linked to nucleosides and they can form intermolecular non-covalent interactions hence the biodegradability asset of this gel [92]. An example of hydrogel forming nucleolipids-based LMWG are glycosyl-nucleoside-lipids (GNLs) owing to their intrinsic supramolecular properties [98]. These types of molecules are amphiphilic, given their particular structure consisting of a hydrophobic tail and a hydrophilic head group, giving upward thrust to their surfactant properties and conjointly their ability to self-assemble into nanostructures [98]. In view of their transfection capabilities, they are valued in biomedical research for the delivery of nucleic acids into cells [99].

Another interesting molecular structure for gelators design is the bola-amphiphile structure. The self-assembling features of bolas have been described for decades now [100] and inspired many bola-based gelators [101,102,103]. From this design, further other derivatives have shown their potential such as glycosyl-nucleoside-bola-amphiphiles (GNBAs). They consist of the same moieties of GNL arranged in a bola structure: the two polar heads (glycosylated nucleobases) at each end of a hydrophobic chain (lipidic chain)[80]. Their interesting self-assembly characteristic that bola-amphiphiles hold allows the creation of a unique architectural hydrogel that features fast gelation kinetics, high elastic moduli, thixotropic and thermal reversibility properties [98,104]. 

Synthetic hydrogels, prominently amphiphilic molecules based gels, are used in a variety of applications such as nano-material synthesis [105] and more importantly in the biomedical field for tissue engineering, wound healing or drug delivery [106,107,108].

## 3. Cancer Applications of Hydrogels

Cancer cell lines are considered today as significant in vitro models in the medical field primarily due to their distinctive potential to produce an unlimited amount of biological material. The conception of databases that describe thoroughly the molecular and cellular diversifications of cancer cells such as mutations and difference in gene expression, ease the selection process of the desired cell type [109].

Unlike normal cell lines that present a short life span, cancer cell lines can be passaged with a low mortality rate. Owing to their anchorage-independent feature, they are convenient candidates for 3D culture. They are often observed to have a small size, more rounded with a higher nucleus/cytoplasm ratio by comparison to normal cells. Moreover, they have low serum dependency, as well as a higher growth rate and a higher ability of cloning than that of normal cells observed during toxicology assays [110]. 

Previous studies for example proved that cancer lines have a greater potential in drug-induced liver injury toxicity assessment, compared to conventional human hepatocytes. They also preserved some toxicity-related pathways throughout different assay systems, such as peroxisome proliferator-activated receptor pathway and fatty acid-related pathways [111].

Consequently, cancer cell lines are commonly used during drug design and treatment optimization [112,113,114,115,116], for personalized medicine using patient derived tumor cells [117] and in vitro toxicity testing of physico-chemical contaminants. In particular, recently many studies demonstrated the use of cancer cells as support for nanoparticles (NPs) toxicity assays [118,119]. Nanoparticles are proven to have a certain impact on cancer development, thus its toxicity could be correlated to the severity of the situation. [120,121]

Hydrogels retained historically a major application in biomedical research in particular tumor modeling in vitro [96,122].

Cancer research was subjected over the years to a load of optimizations and enhancement in experimentations. Scientists have gone from theories, to practical examination of cancer cells regarding their behavior, progression and reaction vis-a-vis therapy approaches. Cell culture was and remains until this day, the foundation of the development of today’s well-known cancer therapies. Without cell culture assays, we would not have understood how cancer cells answer when exposed to chemotherapy or radiotherapy. We would not have understood the concept of metastasis, therapeutic resistance and the emersion of therapeutic multi-resistance. The emergence of 3D culture helped us overcome many 2D culture barriers and took cancer research to a completely new dimension. The ECM-mimicking network created inside the biomimetic hydrogels allows us to recreate the physiological microenvironment in which cells grow and develop inside the human body. Accordingly, hydrogels became a more suitable microenvironment, in which, 3D culture of hosted cancer cells leads the cells to behave in a physiological manner. This behavior of cancer cells drives toward a more realistic tumor progression. Meaning, the cells are going to assemble inside the nano-fibrous network, and will proliferate and differentiate as they would in the ECM. Depending on the nature of the polymer used to create the hydrogels, the cells will eventually create their own ECM and their own tumor niche [123]. In this kind of biomaterials, the assessment of tumor progression can be done from start to end because analysis of cell behavior, using different biological assays, can be done at any given time. It is important to understand how cancer cells begin their journey into tumor construction, so that innovative early-stage treatments can be conceived. 

On another hand, unlike other 3D systems, these kinds of hydrogel networks allow the creation of multicellular tumor spheroids (MCTS) (Figure 2D). MCTS are solid cancer models that are created from a small number of seeded cells in a complex architecture. They represent solid tumors found inside the human body such as glioblastoma. This kind of cancer model is very particular, very interesting and not easy to obtain [124].

As their name implies, MCTS are a well-constructed series of cell layers forming a necrotic center, followed by a layer of quiescent cells and finally peripheral proliferative cells. An oxygen and nutrient gradient is created from the tumor surface to the core. This structure is uniform to the solid tumors observed in vivo. The conception of this structure culturing cancer cells in hydrogels, promote many possibilities of cancer therapy trials. The results using this in vitro model are similar when applied to the in vivo tumors [6,11,125,126]. 

Nowadays, in the field of drug discovery research, MCTS have proven efficient for all-inclusive drug penetration, efficacy and discharge [126]. Owing to their architectural structure, MCTS showed a higher resistance for antineoplastic agents compared to monolayer 2D cells. The reduced number of cells that are exposed to the anticancer drug in 3D MCTS similar to a solid tumor in vivo, and the highly necrotic core of the MCTS explain this high resistance to cancer therapy trials. In addition, the establishment of an ECM surrounding the MCTS can limit the drug penetration by comparison to a 2D culture. Thus, this is a clarification of the failed attempts of cancer treatment in vivo [127]. Many method articles have exposed detailed protocols for a successful production of MCTS inside a 3D microenvironment [128,129].

Radiotherapy assays have been investigated in 3D spheroids to examine the response of the latter to irradiation. Patient derived tumor cells spheroids demonstrated a radiation-dependent reaction representative of a patient’s specific response. Murine cell lines were also used in 3D irradiation approaches, and alongside human cell lines, they showed radio-biologic hypoxia that requires post treatment reoxygenation [117].

Furthermore, chemo-sensitivity analyses were carried out on 3D MCTS, due to its particular architectural structure. Gene expression profiling of these MCTS showed a similar gene expression of survival, proliferation and resistance genes to those of in vivo tumors by comparison to 2D cultures. In addition, the ECM surrounding the MCTS in the 3D systems was shown to play a key role in chemo-resistance, a role that is held by the physiological ECM during chemotherapy [22,112,113]. Other experiments such as combination therapies, chemotherapeutics and drug carriers can also be studied in these in vitro models [114]. For this matter, nano-vectors for drug delivery were investigated in cancer research. They have the ability to retain in their core a therapeutic payload that can be targeted to the tumor niche [115]. Current breakthroughs were documented in the biomedical research and approved by the FDA, consist of therapeutic nanoparticles encapsulated chemotherapy for the treatment of acute myeloid leukemia [116]. 

Two important questions rise at this point. How can the hydrogel matrix’s complex architecture be used in novel assays? Is MCTS’s importance solely restricted to cancer research?

The porous architecture of hydrogel matrix has been subjected to various diffusion studies over the years. For example, diffusion of phage particles for bacterial bio sensing were analyzed in carbohydrate hydrogels [130]. Macromolecules diffusion in hydrogels were also a center of attention [131], especially protein diffusion inside the matrix such as in PEG hydrogels for the design of an artificial cornea material [132] or poly(sulfobetaine methacrylate) hydrogel for biological transport assays in zwitterionic hydrogels [133]. In addition, chemical elements diffusion such as ions were also investigated in a calcium alginate hydrogel-structure [134]. This means that other entities whether they are macromolecules or simply small particles may also have the ability to diffuse through this complex structure hence promoting the establishment of innovative particle, molecule or entity analysis such as toxicity assays of contaminants. Nevertheless, a thorough toxicity analysis remains insufficient without studying the impact of these contaminants on a living system. This is the important role of MCTS being the living system of interest grown inside the hydrogel.

## 4. Nanoparticles Emergence 

Today, with modern technology, the world is witnessing a global emergence of particles of all sorts, notably NPs. This may have an unforeseen impact on humans and ecosystems, becoming an object of particular attention for public health and environmental studies. 

### 4.1. Definition of Nanoparticles

A nanoparticle is characterized based on its size, shape, structure and chemical composition giving it particular personalized properties. Due to its very small size, it exhibits physical and chemical properties different from those of a big sized object of the same matter [135]. With stable material conditions and a low number of atoms, the majority of the atoms of a nanoparticle are present on the outside rather than the inside of the particle itself. This gives particular atomic properties to a nanoparticle [135]. With the smallest observed nanoparticles ranging slightly less than 1 nm and the biggest going to several tens of nanometers, ISO [136] defined nanoparticles as entities having one or several external dimensions at the nanoscale ranging from about 1 to 100 nm in size. 

Nevertheless, another factor to be taken into consideration is the reactivity of a nanoparticle in regard of the environment in which it is found, due to its very large exchange surface with this environment. The latter can cause, among other transformations, agglomeration (reversible) and aggregation (semi-reversible) assemblies of NPs generating larger nanoparticles going up to several hundreds of nanometers; or dissolve NPs leading to smaller ones, or even to their total disappearance in favor of the ions and/or molecules that made them up. The surrounding medium can also modify the composition of the surface of nanoparticles by sorption or desorption, affecting their reactivity and fate [137]. This is the reason why it is fundamentally important to determine and monitor the NPs state before, during and after an experiment that requires their use. 

### 4.2. Nanoparticles Origin and Environmental Dispersion

Nanoparticles that are generated in or migrate to the environment are ubiquitous [138,139,140,141] and found in natural aqueous media where they can cause potential hazards to living species [142,143,144]. They can have natural origins such as forest fires, dust storms and volcanic ash or anthropogenic origins. The latter are man-made and have two classifications [145].

The first of anthropogenic origins are NPs with no prearranged size nor defined chemistry. For example, they can originate from combustion and diesel exhaust. For a matter of fact, NPs released by diesel engine are urban air pollutants of great importance and may possess a compelling threat on public health. The NP modeling showed that, when released from their host medium into the environment, they could have different compositions and properties such as size distribution and state of agglomeration. This implies that these unintentionally discharged NPs have an unforeseen and unpredictable impact on the environment and its inhabitants [146].

In another category, we can find the engineered or manufactured NPs, with a potentially controlled size range, materials and surface composition [145]. For example, silver nanoparticles (Ag-NPs) were shown to exhibit antimicrobial effect making them interesting in medical devices [147]. Titanium (TiO_2_-NPs) and zinc oxide nanoparticles (ZnO-NPs) have photochemical properties that are exploited for UV protection [148,149], photo degradation [150], pigmenting, etc. Iron oxide nanoparticles (IONPs) have magnetic properties that are used in medical imaging and cell targeting [151]. As a result, these nanomaterials are found in many everyday products, such as cosmetics, textiles, food and medical compounds. They are also used in industrial applications, such as in modified concrete [152,153], energy production [154,155], the health field [156,157], etc. In view of their ubiquity in manufactured products, these NPs are inevitably found in the environment via discharges, waste, etc.

### 4.3. Why Are NPs a Center of Attention Today?

Due to their recent growing emergence and environmental dispersion, NPs interactions with human organisms can originate from different sources. 

NPS have witnessed a large spectrum of applications nowadays. One of the most important is their use as a diagnostic tool for example by functionalizing the surface of NPs (e.g., gold NPs) with biological matter such as antibodies that can detect the presence of certain proteins correlated to a given disease [158]. In addition, NPs have become a center of attention because of their magnetic properties, such as iron oxide considered as superparamagnetic NPs. These NPs can serve as materials for cancer treatment and drug delivery systems owing to their controlled orientation and arrangement in a strong magnetic field [159]. These features of NPs make them ideal candidates as thermal mediators in hyperthermia, drug vectors as well as contrast agents administered to patients for magnetic resonance imaging (MRI) or computed tomography [160]. Thus, whether they are natural, incidental or engineered, NPs emergence and contact with living entities are exponentially growing [161].

Thus, it is a crucial issue to assess their physiological impact and especially their cytotoxicity. Until now, the majority of the NPs toxicity assays were conducted in 2D in vitro models [162,163,164,165,166,167,168,169,170] or in vivo models [171,172,173,174,175,176,177]. Accordingly, gaps in the results were found when it comes to comparing nano-toxicity results between in vitro and in vivo. Consequently, developing in vitro models are needed for NPs toxicity assays knowing that very little progress has been registered in the conception of in vitro models to study the distribution and translocation of nanoparticles across different barriers. The development of more specific in vitro models is of upmost importance to understand systemic effects of nanomaterials [15].

### 4.4. In Vitro Cellular Models for Nanoparticles Toxicity Assays

At first, the standard nano-toxicity assays were mostly based on cytotoxicity and geno-toxicity evaluation for nanoparticles uptake by 2D cancer cells. Ag-NPs [167,168,169,178,179,180,181] and TiO_2_-NPs [162,165,174,182,183,184,185,186,187,188] cytotoxicities are now established, with adopted hypothesis of mechanism involving ROS production, organelles inhibition and DNA damage, in a dose-dependent manner. As mentioned above, the use of cancer cell lines for most of these studies, such as HeLa [181,189], Hep2/HepG2 [180,183,187] or PC-3M [184], leads to more reproducible and homogenous results under similar conditions, helping to focus on interactions between NPs and specific type of cells according to the purpose. For instance, because of the potential dispersion of Ag- and TiO_2_-NPs in the respiratory system, studies of their effects were conducted on A549 lung cancer cell line and permit the determination of some adverse effects, from DNA damage to apoptosis [167,186,188]. In the same spirit, the use of immortal skin cell lines such as HaCaT is consistent with the fact that TiO_2_-NPs is found in everyday products such as cosmetics and with the purpose of uncovering their effect on skins [190]. It is noteworthy that TiO_2_-NPs are still considered as inert for animals and humans, even though some advancements are made on their pulmonary effects [191,192]. For other NPs such as gold, the conclusion is still unclear since some studies demonstrated their cytotoxicity [164,193] and other their harmlessness [194,195].

However, some studies showed the potential of 3D culture in assessing the nano-toxicity in a more relevant way (Table 1). Indeed, by recreating cell–cell interactions and tissue-like behavior, the impact of NPs is clearly influenced. In some studies, 2D cell culture were found to overestimate some harming effects of NPs when compared to 3D cell culture [196,197,198,199]. In 3D spheroid of central nervous system cells cultured in suspension in ultra-low attachment (ULA) wells, IONPs (Fe_3_O_4_) have a concentration and time-dependent effects on cell mortality comparable to those measured in vivo [200]. Thus, the cell mortality seems attenuated compared to 2D cell culture results [201]. Same results were previously obtained in IONPs toxicity assessment in 3D culture of other cell type, with a higher toxicity in 2D monolayer compared to 3D [197].

Relatively, cell sensitivity to nanoparticles is 3D environment dependent. Some 3D cultured cells can be more sensitive to NPs effect, as shown using human lung carcinoma spheroids developed in ULA wells [202]. They displayed a higher sensitivity regarding ZnO-NPs compared to 2D cell monolayer. This study also showed that TiO_2_-NPs induce a minor decrease in cell viability in 3D, whereas they do not exhibit any toxicity in 2D. 3D cells cultured in alginate matrix are more sensitive to IONPs used as nano-carrier for drug delivery and that coating NPs could enhance their biocompatibility [203]. However, 3D cells cultured on agar plate, in which the architecture is less complex, are less sensitive to nano-toxic effects in general, but some inflammatory responses were found to be higher in 3D cells exposed to ZnO-NPs by comparison to 2D [204].

The difference in NPs’ impact between 2D and 3D cell culture is explainable by many considerations. As previously presented, the spatial cell organization is crucial for an extended recovery of molecular and biochemical mechanisms of the cell in in vitro conditions, such as cell behavior when exposed to exogenous factors. Moreover, cell clustering will influence the NPs penetration and thus their hazardous effect. The fact that some 3D models tended to reproduce in vivo results is also a promising clue of their legitimate use [205,206].

A 3D in vitro model is considered as a bio-indicator when used for measuring nano-toxicity. The 3D systems used in these studies demonstrated primary behavior of 3D spheroids when put in contact with nanoparticles. Taking this into account, an upgrade in the implementation of these experiments can render the nano-toxicity assays more predictive and reproducible. 

## 5. Conclusions and Perspectives

Over the recent years, 3D cell culture demonstrated great capacities in cancer research, regenerative medicine and tissue engineering. 3D culture can be produced using either scaffold-free systems such as spheroids and organoids, or scaffold-based systems like solid scaffolds or hydrogels.

In this matter, hydrogels displayed abilities to overcome many limitations that other types of 3D culture impose on researchers such as biodegradability, biocompatibility and most importantly, its inner matrix consists of a complex structure that mimics the physiological ECM hence getting us closer to human conditions. LMWG based hydrogel are capable of producing multicellular tumor spheroids with an initial low-density cell seeding. More importantly, owing to its high reproducibility, an LMWG-based hydrogel can ensure a reproducible production of MCTS, an important feature for reliable results concerning any type of imminent experiments. This in vitro model still manifests today major importance in cancer research in regards of understating tumor progression and therapeutic resistance. However, due to its reproducibility and long cancer cell life span, it could also be used as a bio-indicator to assess the toxicity of chemical elements, molecules or particles that we may encounter in the environment that we live in.

Today, with the emergence of nanoparticles of many sorts, the implementation of a reproducible in vitro model that can host NPs will allow the investigation of their impact and inevitably their burden on public health. 

Based on the previously established NPs toxicity trials, a guideline for the anticipated experiments can be suggested in an attempt to get reproducible and comparable results, more representative of the real-life toxicity of NPs. The guideline consists of choices and alterations to be taken into account on different key points.

First, the choice of the cell line that will be the biological matter of interest is primordial. As described before, the majority of the toxicity experiments are done on cancer cell lines and for all the right reasons. Cancer cell lines are and will always be ideal candidates for such experiments. However, the choice of the cancer cell line is correlated with the type of toxicity in hand. Meaning if we are studying nano-toxicity in food products, the cancer line could be colorectal cancer line, that of the digestive tract. If the study concerns aerial toxicity, the cancer model could be pulmonary cancer line, that of the respiratory tract. These cell lines grown in a 3D system will produce MCTS.

We would like to restate that one of the key points of a successful and repeatable toxicity experiment is the repeatability of the MCTS itself. However, the majority of cancer cell lines derived from solid tumors such as glioblastoma present a high heterogeneity that can compromise the standardization of the MCTS. For this reason, we recommend exploiting cell sorting techniques to sort specific subpopulations from heterogeneous populations. The use of these subpopulations will ensure the standardization of the in vitro cellular model [10,207]. 

Second, at the light of the previously discussed 3D systems, an LMWG based hydrogel, which is a biomimetic synthetic scaffold, is a more fitting candidate that offers the ideal architecture for 3D culture as well as molecules and nanoparticle diffusion. In a system such as the latter, the discrimination between NPs and colloidal natural entities becomes easier [208]. These gels should be well characterized and optimized so properties such as stiffness and structural stability resemble that of the ECM hosting the cells inside the human body [209]. Moreover, since the biophysical properties of the cellular microenvironment influence the cellular behavior, such as cell differentiation, morphology and proliferation, gel characterization should be done in parallel with biological characterization of the encapsulated cells in order to relate the 3D system to the cellular model features. Then, the reproducible standardized MCTS grown inside these gels can serve as predictive models for quantifying NPs effects on cell functionalities. Several mathematical equations were conceived to better quantify these responses on the following hallmarks. The binding of the NPs on cell surface by means of ligand-receptor association, the occurrence of endocytosis meaning the uptake of NPs inside the MCTS cells and vice versa the expulsion of NPs by exocytosis and the interaction of NPs and the ECM will influence a specific movement of the NPs in the interstitial space of the MCTS. An understanding of these responses will give a clearer insight on NPs behavior in a human-mimicking microenvironment [210,211]. In addition, the entry of NPs into solid tumors can serve as vectors for potential treatment of these cancers. [212]

**Figure 4:**
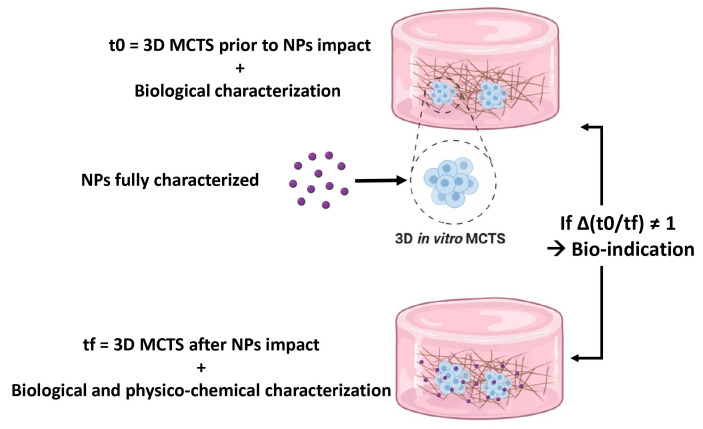
Initial state t0 vs. final state tf of the MCTS. In order to assess the effectiveness of the bio-indicator, a comparison between t0 and tf is inevitable for the understanding of the impact of NPs on the living in vitro 3D model.

Third, prior to studying the impact of an exogenous factor on a biological entity, the latter should be characterized beforehand. This means that before the NPs are put in contact with the 3D MCTS, the latter must undergo biological characterizations. These characterizations that define the initial state of the MCTS (t0) (Figure 4), will give us information about the structure of the MCTS (the three previously described layers), the state of differentiation of the cells, the state of viability and proliferation of the cells, etc.

Fourth, manufactured nanoparticles disseminated in the environment are emerging pollutants present in the air, water and soil and likely to contaminate and affect the quality of the environment, water resources and agricultural production. It is important to evaluate, in a thorough manner, the characteristics of these entities of interest: their multidimensional state significantly the size of the NPs, their morphological state, chemical composition, their concentration, their size distribution and state of aggregation (homo or hetero aggregation). This information should be acquired throughout the experiment for a controlled experiment and in order to correlate the given results with a given environment. Most of the size characterizations are done by transmission electron microscopy (TEM) [16] that provides an effective representation of the spheroid ultra-structure on a nano scale and dynamic light scattering (DLS) among zeta potential characterization [213]. Taking into account the NPs surrounding environment during the experiment should be a reflection of the environment in which given NPs were found and which motivated the study. For example, assessing the effect of NPs on gut environment needs to be preceded by characterization of these NPs in a medium having at least similar acidity and ionic strength, also their interaction with the components of culture media. FBS-supplemented (fetal bovine serum) cell medium contains many factors that can influence NPs behavior, such as protein adsorption on NPs surface that can change their surface charge [214,215,216], influence their aggregation and eventually hinder their harmful effect. Moreover, the influence of the time point of NPs exposure needs to be taken into account to define a representative 3D model for in vitro nano-toxicity assay [202].

Until this day, quantitative information is still needed regarding the concentration-dependent size distribution of the particles at any given time. We must insist that knowing the nanoparticle physico-chemical state before, during and after the experiment is the only true indicator of the cross-effect of these NPs and their given environment. Along with the analytical run, sample preparation is also a crucial step. Data is regularly published on the subject. However, hardly any of these publications have validated the approach and/or tested it in biological or environmental monitored system. 

Associated with sample preparation, the preparation of the study medium, typically for hosting NPs and biological cells, or for studying the behavior of NPs in environments simulating the environment remains a totally open question to this day.

For this in vitro model to be considered as a bio-indicator, NPs distribution and time points studies as well as series of biological analysis must be performed on the in vitro cellular model subsequent to NPs contact, which constitutes the final state of the MCTS (tf) (Figure 4 below).

Which brings us to the fifth target, the time points of NPs exposure to the MCTS inside the hydrogel. The use of hydrogel as a cell culture scaffold leads to thinking about how to expose encapsulated cells to external factors. The fibrillar architecture and network permit the diffusion of molecules and NPs through the interfibrillar spaces, such as molecules and NPs. Although, the diffusion kinetics of these entities from outside to inside the gel have to be determined in order to take into consideration the delay between their administration and their effective uptake by the cells. We must also ensure that the administered factors are homogeneously diffused in the whole volume of the hydrogel. It is noteworthy that this concerns NPs as well as reagents for biological analysis. The 2D cell culture cannot give this kind of information since it lacks intercellular organization, so 3D cell culture is a suitable model to study the impact on NPs on multicellular formation and physiology. Using a 3D system such as ULA to study the spheroid development upon NPs exposure facilitates NPs uptake [202], but as previously mentioned, the diffusion kinetic through the hydrogel is also a parameter to be considered. Finally, in a context of NPs dissemination in the environment or human being exposed to NPs from everyday products or professional activity, the frequency of NPs uptake by 3D cultured cells must be determined according to the issue (e.g., chronic or acute exposure). This also brings another layer of complexity to be treated, for instance knowing how the hydrogel reacts to long term exposure to NPs or if there is some architecture disruption upon high dose on short term. In all cases, the interaction between NPs and the 3D-cells system must be fully explored by integrating the timescale that will be considered for the experiment, for the sake of results relevance. 

Finally, just like for the initial state (t0), a series of biological analysis must be performed at the final state (tf) of the in vitro cellular model. For example, cell viability is one of the first factors investigated during toxicology assays to assess the impact of entities on the life cycle of the cellular model. For this matter, a tetrazolium-based colorimetric such as MTT, WST-1 and MTS is used to assess the cell metabolic activity and consequently their viability [118,217,218]. Cell membrane integrity can also be evaluated using lactate dehydrogenase (LDH) assay [118].

Notably, using a hydrogel matrix might influence the diffusion and the dilution of reagents used in cell viability tests, so it has to be taken into account when deciding about the conditions of these assays [219]. Since cell death can either occur by apoptosis, which is a programed regulated cell death, or necrosis, which is an incidental cell death due to non-physiological factors such as infection or particle impact; discriminating between both types is inevitable. Scanning and flow cytometry are the perfect techniques that allow single-cell study and that are able to discriminate between both forms of cell death [220]. The cell inflammatory response can also be induced by NPs and has to be examined using ELISA targeting inflammatory biomarkers in the likes of IL-8, IL-6 and tumor necrosis factor [118]. Oxidative injury is also a significant biological incident that needs to be assessed by determining the levels of total antioxidant capacity (TAC) and total oxidative stress (TOS) [221], using ELISA or spectrophotometric methods [222].

We also recommend a genotoxicity study using a bioluminescent whole-cell bioreporter, such as E-coli bioreporter, for quantitative evaluation of the DNA damage induced by the NPs and other mutagenicity such as clastogenic and aneugenic effects and chromosomal structure abnormalities. Furthermore, assessing organelles dysfunction can also be predictive of NPs impact on cells physiological function by potentially inducing cell autophagy. For example, examining mitochondrial dysfunction by studying ATP production for respiratory control ratio.

An extra advantage for using cancer models is assessing tumor aggressiveness and progression following NPs impact. These observations can be done by studying the state of differentiation of the cells forming the MCTS and by doing cell invasion assays that are naturally correlated with metastatic features. Such studies can be reflective of a potential cancer development subsequent to NPs interaction with human organisms. 

The production of repeatable MCTS, by the means of 3D culture of cancer lines inside hydrogels, and the characterization of NPs and control of their spatiotemporal behavior, consist of the ideal cartography of a bio-indicator for nanoparticle toxicity assays. The biological analysis of MCTS at tf will inform us on the cellular behavior and state subsequent to their exposure to NPs.

Thus, the study of Δ(t0/tf) (Figure 4) is informative of the difference of cellular properties before and after NPs impact. If Δ(t0/tf) truly demonstrates a significant difference, then this 3D in vitro cellular model can be considered as an ideal successful bio-indicator. This will allow the standardization of the 3D system in a global manner that might help in obtaining results that are more concordant.

However, some of the targeted information should be unified in an intra and inter-laboratories manner. What is the best cartography of this 3D system? What are the most relevant tests to be performed in order to highlight a significant impact? 

## Figures and Tables

**Table 1:** Brief of nanoparticles (NPs) toxicity in 2D vs. 3D: cell line/NPs used/3D system/analysis.

NPs	NP Size (nm)	NP Coating	NP Concentration	Cell Line	3D System	Cytotoxicity Assays	Cytotoxicity and Comparison to 2D Cell Culture	Ref.
ZnO	24; 56; 87	-	10; 25; 50; 75; 100 µg mL^−1^	Caco-2 (colorectal adenocarcinoma )	Agarose gel	ROS expression (CellROX orange reagent); measurement of pro-inflammatory cytokines (IL-8 and IL-1β); cell proliferation (PicoGreen); modes of cell death (AnnexinV_FITC/PI)	Increased ROS expression; size-independent toxicity; decreased DNA amount at high concentrations; cell death: apoptosis in 3D vs. necrosis in 2D; less toxic in 3D than in 2D	[187]
	222.0 (in water); 142.2 (in FBS-DMEM)	-	31.5; 125; 500; 1000 µM	SW480 and NCM460 (colorectal cancer)	Agarose gel	Mode of cell death (annexinV-PI); Immunofluorescence staining of NF-κB; metabolic activity (MTT method)	Increased ROS expression in NCM460 cells upon NPs uptake but no ROS increased in 3D SW480; higher basal ROS expression in 3D SW480 than in 2D; ROS-independent inflammatory response increased in both cell lines in 2D and 3D; 3D is more resistant to DNA damage than 2D	[192]
ZnO TiO_2_	ZnO: 41 ± 5 (in water); 190 ± 3 (in DMEM); 106 ± 11 (in standard culture medium); TiO_2_: 79 ± 25 (in water); 142 ± 29 (in DMEM); 118 ± 28 (in culture medium)	-	0.5–2.5 µg mL^−1^	A549 (human lung carcinoma); NIH-3T3 (mouse fibroblasts)	ULA well plates (spheroids)	Cell viability (CellTiter-Blue viability test; reduction of blue resazurin to purple resofurin monitored by fluorescence); (CellTiter-Glo luciferase reaction in presence of Mg/ATP/oxygen; monitored by luminescence); cell morphology (microscopy)	ZnO: similar toxicity in NIH-3T3 in 2D and 3D;A549 more sensitive in 3D than in 2D; both toxicity displayed in concentration-dependent manner; TiO_2_: small toxic effect in 3D; no toxicity in 2D	[190]
TiO_2_ Au Ag	Au “15 nm”: 51 ± 6; Au “80 nm”: 116 ± 5; TiO_2_: 896 ± 133; Ag: 120 ± 4 (in DMEM)	Au: phosphine TiO_2_: PVP	1.25–625 µg cm^−2^	Caco2 (colorectal adenocarcinoma) mono-cultured of co-cultured with THP-1 (human macrophages) and MUTZ-3 (human dendritic cells)	Collagen hydrogel	Inflammatory response (IL-8); cell viability (alamar blue assay; LDH assay)	No impact of Au on cell viability or inflammation in Caco2 mono-culture but slight increase of IL-8 in co-cultured cells; mono-cultured cells more sensitive to Ag; inflammatory response from Ag at any concentration in co-culture; no toxic or inflammatory effect for TiO_2_ in both mono- and co-cultured cells	[184]
Ag ZnO SiO_2_	Ag: 6.873 ± 3.330 (in water); 6.887 ± 4.176 (in cell culture medium); ZnO: 43.58 ± 9.113 and 292 ± 42.50 (in water); 134.7 ± 108.2 and 3902.0 ± 1119.0 (in cell culture medium); SiO_2_: 30.99 ± 20.0 (in water); 41.65 ± 22.09 (in cell culture medium)	-	Ag: 5–50 µg mL^−1^; ZnO: 10–120 µg mL^−1^; SiO_2_: 0–2000 µg mL^−1^	HepG2 (hepatocarcinoma)	Gelatin; collagen hydrogel; Matrigel	Metabolic activity (albumin and urea assays); cell viability (trypan blue and alamar blue); colorimetric cell proliferation assay (CellTiter96); morphology (microscopy)	3D show less toxicity than 2D; FBS hinder toxic effect of NPs but no change cell survival; Increase spheroids disaggregation at higher frequency of low concentration of NPs uptake compared to lower frequency of high concentration	[204]
CdTe Au	CdTe: 2.4–6; Au: 3.5 ± 0.7 (in citrate); 5.5 ± 0.6 (in CTAB^1^)	CdTe-NPs: L-cysteine monolayer; Au-NPs: citrate/CTAB	CdTe: 10 µg mL^−1^; Au: 98.5 µg mL^−1^	HepG2 (hepatocarcinoma)	Polyacrylamide hydrogel	Cell morphology (SEM; light microscopy); membrane integrity; metabolic activity (LDH and MTT methods); cell death mechanism (caspase assay for apoptotis; LDH for necrosis); live/dead assays (confocal microscopy)	Drop of cells metabolism and change in phenotype upon CdTe and CTAB-Au uptake; no toxicity reported for citrate-Au; less toxic in 3D than in 2D	[194]
IO	fluidMAG-D: 150; fluidMAG- PEI: 100; fluidMAG- CMX: 150	D: neutral starch PEI: cationic polyethyleneimine: anionic carboxymethydextran	25 µg cm^−2^	HBMEC (Human brain microvascular endothelial cells)	Spheroids suspension in inverted plate	Real-time cell analysis based on electronic impedance measurement via electrode at the bottom of wells; immunoblotting (Akt signalling); stained cell observation (confocal microscope)	Increased Akt activation in 2D vs. 3D upon NPs uptake; NPs distribution is coating charge-dependent; cell death more pronounced in 2D vs. in 3D	[185]
	Fe_3_O_4_: 48.7 (in sodium citrate); 10; 25; 70; 700 (in cell culture medium)	Polyvinylpyrrolidone (PVP)	0.1–25 µg mL^−1^ (long term exposure); 1–100 µg mL^−1^ (short term exposure)	D384 (astrocyte); SH-SY5Y (neuroblastoma)	ULA well plates (spheroids)	Cell viability (Trypan blue); cell morphology (microscopy)	Concentration-dependent cell mortality and spheroid disaggregation; at highest concentration: 50% and 34% cell viability decrease of D384 and SH-SY5Y respectively in 3D vs 75% and 45% in 2D	[188,193]
	5 and 30	Dextran; PEG or no coating	100; 250; 500 µg mL^−1^	PAEC (Porcine aortic endothelial cells)	Alginate hydrogel	Cell viability (live/dead assay; alamar blue assay); ROS level; cell shape (actin cytoskeleton labeled and microscopy)	Bare NPs decrease cell viability and increase ROS expression in dose-dependent manner; any coating reduces cytotoxicity and ROS expression with no effect of the size in both 2D and 3D; bare NPs more toxic at low concentration in 3D vs. 2D	[184]
PELGA ^2^	PELGA10: 79.2 ± 5.7; PELGA20: 90.5 ± 5.0; PELGA40: 175.8 ± 4.3	-	2 µg mL^−1^	HeLa (human epithelioid cervix carcinoma)	Collagen hydrogel	Cell viability (alamar blue assay); cell morphology (microscopy)	Sub-100 nm NPs more internalized and toxic in 3D than in 2D	[177]
Polymicells drug nanocarrier	151.9	-	100 µg mL^−1^	95-D (lung cancer); U87 (glioblastoma); HCT 116 (colorectal cancer)	Collagen hydrogel	Metabolic activity (MTT method); cell morphology (microscopy)	Attenuation of antitumoral effect and drug sensitivity in 3D vs. in 2D.	[186]

^1^ cetyltrimethylammonium bromide. ^2^ poly(D,L-lactic-co-glycolic acid) (PLGA)–block–poly(ethylene glycol) (PEG). Numbers 10, 20 and 40 indicate the concentration (mg ml^−1^) of PEG-PLGA used during NPs preparation.

## References

[1] Duval K., Grover H., Han L.-H., Mou Y., Pegoraro A.F., Fredberg J., Chen Z. (2017). Modeling Physiological Events in 2D vs. 3D Cell Culture. Physiology.

[2] Ravi M., Paramesh V., Kaviya S., Anuradha E., Solomon F.P. (2015). 3D Cell Culture Systems: Advantages and Applications. J. Cell. Physiol..

[3] Muthuswamy S.K. (2011). 3D culture reveals a signaling network. Breast Cancer Res..

[4] Sittampalam S., Eglen R., Ferguson S., Maynes J.T., Olden K., Schrader L., Shelper T., Ferrer M. (2015). Three-Dimensional Cell Culture Assays: Are They More Predictive of In Vivo Efficacy than 2D Monolayer Cell-Based Assays?. Assay Drug Dev. Technol..

[5] Edmondson R., Broglie J.J., Adcock A.F., Yang L. (2014). Three-Dimensional Cell Culture Systems and Their Applications in Drug Discovery and Cell-Based Biosensors. Assay Drug Dev. Technol..

[6] Langhans S.A. (2018). Three-Dimensional in Vitro Cell Culture Models in Drug Discovery and Drug Repositioning. Front. Pharmacol..

[7] Cekanova M., Rathore K. (2014). Animal models and therapeutic molecular targets of cancer: Utility and limitations. Drug Des. Dev. Ther..

[8] Voskoglou-Nomikos T., Pater J.L., Seymour L. (2003). Clinical Predictive Value of the in Vitro Cell Line, Human Xenograft, and Mouse Allograft Preclinical Cancer Models. Clin. Cancer Res..

[9] Knight E., Przyborski S. (2015). Advances in 3D cell culture technologies enabling tissue-like structures to be created in vitro. J. Anat..

[10] Bielecka Z.F., Maliszewska-Olejniczak K., Safir I.J., Szczylik C., Czarnecka A.M. (2017). Three-dimensional cell culture model utilization in cancer stem cell research. Biol. Rev..

[11] Nguyen H.T.-L., Nguyen S.T., Van Pham P. (2016). Concise Review: 3D cell culture systems for anticancer drug screening. Biomed. Res. Ther..

[12] Till U., Gibot L., Vicendo P., Rols M.-P., Gaucher M., Violleau F., Mingotaud A.-F. (2016). Crosslinked polymeric self-assemblies as an efficient strategy for photodynamic therapy on a 3D cell culture. RSC Adv..

[13] Mak I.W., Evaniew N., Ghert M. (2014). Lost in translation: Animal models and clinical trials in cancer treatment. Am. J. Transl. Res..

[14] Hoarau-Véchot J., Rafii A., Touboul C., Pasquier J. (2018). Halfway between 2D and Animal Models: Are 3D Cultures the Ideal Tool to Study Cancer-Microenvironment Interactions?. Int. J. Mol. Sci..

[15] Donaldson K., Borm P.J., Castranova V., Gulumian M. (2009). The limits of testing particle-mediated oxidative stress in vitro in predicting diverse pathologies; relevance for testing of nanoparticles. Part. Fibre Toxicol..

[16] Mapanao A.K., Voliani V. (2020). Three-dimensional tumor models: Promoting breakthroughs in nanotheranostics translational research. Appl. Mater. Today.

[17] Antoni D., Burckel H., Josset E., Noel G. (2015). Three-Dimensional Cell Culture: A Breakthrough in Vivo. Int. J. Mol. Sci..

[18] A Multicellular 3D Heterospheroid Model of Liver Tumor and Stromal Cells in Collagen Gel for Anti-Cancer Drug Testing—ScienceDirect. https://www.sciencedirect.com/science/article/abs/pii/S0006291x13004026?via%3Dihub.

[19] Soares C.P., Midlej V., De Oliveira M.E.W., Benchimol M., Costa M.L., Mermelstein C. (2012). 2D and 3D-Organized Cardiac Cells Shows Differences in Cellular Morphology, Adhesion Junctions, Presence of Myofibrils and Protein Expression. PLoS ONE.

[20] Bokhari M., Carnachan R.J., Cameron N.R., Przyborski S.A. (2007). Culture of HepG2 liver cells on three dimensional polystyrene scaffolds enhances cell structure and function during toxicological challenge. J. Anat..

[21] Multi-Channel 3-D Cell Culture Device Integrated on a Silicon Chip for Anticancer Drug Sensitivity Test—ScienceDirect. https://www.sciencedirect.com/science/article/pii/S0142961204005046?via%3Dihub.

[22] Fang Y., Eglen R.M. (2017). Three-Dimensional Cell Cultures in Drug Discovery and Development. Slas Discov. Adv. Life Sci. R. D.

[23] Vinci M., Gowan S., Boxall F., Patterson L., Zimmermann M., Court W., Lomas C., Mendiola M., Hardisson D., Eccles S.A. (2012). Advances in establishment and analysis of three-dimensional tumor spheroid-based functional assays for target validation and drug evaluation. BMC Biol..

[24] Hollister S.J. (2005). Porous scaffold design for tissue engineering. Nat. Mater..

[25] Osteochondral Tissue Engineering SpringerLink. https://link.springer.com/book/10.1007%2F978-3-319-76711-6.

[26] Matrice Poreuse et Culture de Cellules Primaires: Un Même Concept Pour La Reconstruction Cutanée et Cornéenne—ScienceDirect. https://www.sciencedirect.com/science/article/abs/pii/S0369811408001272?via%3Dihub.

[27] Jiang S., Lyu C., Zhao P., Li W., Kong W., Huang C., Genin G.M., Du Y. (2019). Cryoprotectant enables structural control of porous scaffolds for exploration of cellular mechano-responsiveness in 3D. Nat. Commun..

[28] Alghuwainem A., Alshareeda A.T., Alsowayan B. (2019). Scaffold-Free 3-D Cell Sheet Technique Bridges the Gap between 2-D Cell Culture and Animal Models. Int. J. Mol. Sci..

[29] Smith I.O., Liu X.H., Smith L.A., Ma P.X. (2009). Nanostructured polymer scaffolds for tissue engineering and regenerative medicine. Wiley Interdiscip. Rev. Nanomed. Nanobiotechnol..

[30] The Synergy of Scaffold-Based and Scaffold-Free Tissue Engineering Strategies: Trends in Biotechnology. https://www.cell.com/trends/biotechnology/fulltext/S0167-7799(18)30026-X?_returnURL=https%3A%2F%2Flinkinghub.elsevier.com%2Fretrieve%2Fpii%2FS016777991830026X%3Fshowall%3Dtrue.

[31] Youn B., Sen A., Behie L., Girgis-Gabardo A., Hassell J. (2006). Scale-Up of Breast Cancer Stem Cell Aggregate Cultures to Suspension Bioreactors. Biotechnol. Prog..

[32] Lancaster M.A., Knoblich J.A. (2014). Organogenesis in a dish: Modeling development and disease using organoid technologies. Science.

[33] Cramer S.M., Larson T.S., Lockett M.R. (2019). Tissue Papers: Leveraging Paper-Based Microfluidics for the Next Generation of 3D Tissue Models. Anal. Chem..

[34] Luo H., Cha R., Li J., Hao W., Zhang Y., Zhou F. (2019). Advances in tissue engineering of nanocellulose-based scaffolds: A review. Carbohydr. Polym..

[35] Tandon S., Kandasubramanian B., Yakout S.M. (2020). Silk-based Composite Scaffolds for Tissue Engineering Applications. Ind. Eng. Chem. Res..

[36] Chirani N., Gritsch L., Motta F.L., Fare S. (2015). History and Applications of Hydrogels. J. Biomed. Sci..

[37] Cell Encapsulation Using Biopolymer Gels for Regenerative Medicine SpringerLink. https://link.springer.com/article/10.1007%2Fs10529-010-0221-0.

[38] Hydrogels for Biomedical Applications—ScienceDirect. https://www.sciencedirect.com/science/article/abs/pii/S0169409X01002393?via%3Dihub.

[39] Nelson S.R., Zhang C., Roche S., O’Neill F., Swan N., Luo Y., Larkin A., Crown J., Walsh N. (2020). Modelling of pancreatic cancer biology: Transcriptomic signature for 3D PDX-derived organoids and primary cell line organoid development. Sci. Rep..

[40] Kalabis J., Wong G.S., Vega M.E., Natsuizaka M., Robertson E.S., Herlyn M., Nakagawa H., Rustgi A.K. (2012). Isolation and characterization of mouse and human esophageal epithelial cells in 3D organotypic culture. Nat. Protoc..

[41] Nisbet D.R., Forsythe J.S., Shen W., Finkelstein D.I., Horne M. (2008). Review Paper: A Review of the Cellular Response on Electrospun Nanofibers for Tissue Engineering. J. Biomater. Appl..

[42] Kopeček J. (2002). Swell gels. Nat. Cell Biol..

[43] Draper E.R., Adams D.J. (2017). Low-Molecular-Weight Gels: The State of the Art. Chem.

[44] Buxboim A., Ivanovska I.L., Discher D.E. (2010). Matrix elasticity, cytoskeletal forces and physics of the nucleus: How deeply do cells ‘feel’ outside and in?. J. Cell Sci..

[45] Zhu J., Marchant R.E. (2011). Design properties of hydrogel tissue-engineering scaffolds. Expert Rev. Med. Devices.

[46] Osswald A., Hedrich V., Sommergruber W. (2019). 3D-3 Tumor Models in Drug Discovery for Analysis of Immune Cell Infiltration. Methods Mol. Biol..

[47] Zou L., Luo Y., Chen M., Wang G., Ding M., Petersen C.C., Kang R., Dagnaes-Hansen F., Zeng Y., Lv N. (2013). A simple method for deriving functional MSCs and applied for osteogenesis in 3D scaffolds. Sci. Rep..

[48] Gyles D.A., Castro L.D., Silva J.O.C., Ribeiro-Costa R.M. (2017). A review of the designs and prominent biomedical advances of natural and synthetic hydrogel formulations. Eur. Polym. J..

[49] Baharvand H., Hashemi S.M., Ashtiani S.K., Farrokhi A. (2006). Differentiation of human embryonic stem cells into hepatocytes in 2D and 3D culture systems in vitro. Int. J. Dev. Biol..

[50] Glowacki J., Mizuno S. (2008). Collagen scaffolds for tissue engineering. Biopolymers.

[51] Sakai S., Hirose K., Taguchi K., Ogushi Y., Kawakami K. (2009). An injectable, in situ enzymatically gellable, gelatin derivative for drug delivery and tissue engineering. Biomaterials.

[52] Awad H.A., Wickham M.Q., Leddy H.A., Gimble J.M., Guilak F. (2004). Chondrogenic differentiation of adipose-derived adult stem cells in agarose, alginate, and gelatin scaffolds. Biomaterials.

[53] Willerth S.M., Arendas K.J., Gottlieb D.I., Sakiyama-Elbert S.E. (2006). Optimization of fibrin scaffolds for differentiation of murine embryonic stem cells into neural lineage cells. Biomaterials.

[54] Osathanon T., Linnes M.L., Rajachar R.M., Ratner B.D., Somerman M.J., Giachelli C.M. (2008). Microporous nanofibrous fibrin-based scaffolds for bone tissue engineering. Biomaterials.

[55] Mauney J.R., Nguyen T., Gillen K., Kirker-Head C., Gimble J.M., Kaplan D.L. (2007). Engineering adipose-like tissue in vitro and in vivo utilizing human bone marrow and adipose-derived mesenchymal stem cells with silk fibroin 3D scaffolds. Biomaterials.

[56] Wang Y., Kim H.-J., Vunjak-Novakovic G., Kaplan D.L. (2006). Stem cell-based tissue engineering with silk biomaterials. Biomaterials.

[57] Gerecht S., Burdick J.A., Ferreira L.S., Townsend S.A., Langer R., Vunjak-Novakovic G. (2007). Hyaluronic acid hydrogel for controlled self-renewal and differentiation of human embryonic stem cells. Proc. Natl. Acad. Sci. USA.

[58] Chen D., Qu Y., Hua X., Zhang L., Liu Z., Pflugfelder S.C., Li D.-Q. (2017). A hyaluronan hydrogel scaffold-based xeno-free culture system for ex vivo expansion of human corneal epithelial stem cells. Eye.

[59] Kim I.-Y., Seo S.-J., Moon H.-S., Yoo M.-K., Park I.-Y., Kim B.-C., Cho C.-S. (2008). Chitosan and its derivatives for tissue engineering applications. Biotechnol. Adv..

[60] Aisenbrey E.A., Murphy W.L. (2020). Synthetic alternatives to Matrigel. Nat. Rev. Mater..

[61] Ko K.R., Tsai M.-C., Frampton J.P. (2019). Fabrication of thin-layer matrigel-based constructs for three-dimensional cell culture. Biotechnol. Prog..

[62] Dai Y., Siemann D. (2019). C-Src is required for hypoxia-induced metastasis-associated functions in prostate cancer cells. OncoTargets Ther..

[63] Bogacheva M.S., Khan S., Kanninen L.K., Yliperttula M., Leung A.W., Lou Y.-R. (2018). Differences in definitive endoderm induction approaches using growth factors and small molecules. J. Cell. Physiol..

[64] Ribeiro A.J.S., Ang Y.-S., Fu J.-D., Rivas R.N., Mohamed T.M.A., Higgs G.C., Srivastava D., Pruitt B.L. (2015). Contractility of single cardiomyocytes differentiated from pluripotent stem cells depends on physiological shape and substrate stiffness. Proc. Natl. Acad. Sci. USA.

[65] Wimmer R.A., Leopoldi A., Aichinger M., Kerjaschki D., Penninger J.M. (2019). Generation of blood vessel organoids from human pluripotent stem cells. Nat. Protoc..

[66] Talbot N.C., Caperna T.J. (2014). Proteome array identification of bioactive soluble proteins/peptides in Matrigel: Relevance to stem cell responses. Cytotechnology.

[67] Hughes C.S., Postovit L.M., Lajoie G.A. (2010). Matrigel: A complex protein mixture required for optimal growth of cell culture. Proteomics.

[68] Hansen K.C., Kiemele L., Maller O., O’Brien J., Shankar A., Fornetti J., Schedin P. (2009). An In-solution Ultrasonication-assisted Digestion Method for Improved Extracellular Matrix Proteome Coverage *. Mol. Cell. Proteom..

[69] Sionkowska A. (2011). Current research on the blends of natural and synthetic polymers as new biomaterials: Review. Prog. Polym. Sci..

[70] Soofi S.S., Last J.A., Liliensiek S.J., Nealey P.F., Murphy C.J. (2009). The elastic modulus of Matrigel™ as determined by atomic force microscopy. J. Struct. Biol..

[71] Catoira M.C., Fusaro L., Di Francesco D., Ramella M., Boccafoschi F. (2019). Overview of natural hydrogels for regenerative medicine applications. J. Mater. Sci. Mater. Med..

[72] Ruedinger F., Lavrentieva A., Blume C., Pepelanova I., Scheper T. (2015). Hydrogels for 3D mammalian cell culture: A starting guide for laboratory practice. Appl. Microbiol. Biotechnol..

[73] Slaughter B.V., Khurshid S.S., Fisher O.Z., Khademhosseini A., Peppas N.A. (2009). Hydrogels in Regenerative Medicine. Adv. Mater..

[74] Zhao D., Jiang W., Wang Y., Wang C., Zhang X., Li Q., Han D. (2020). Three-Dimensional-Printed Poly-L-Lactic Acid Scaffolds with Different Pore Sizes Influence Periosteal Distraction Osteogenesis of a Rabbit Skull. BioMed Res. Int..

[75] Tibbitt M.W., Anseth K.S. (2009). Hydrogels as extracellular matrix mimics for 3D cell culture. Biotechnol. Bioeng..

[76] Ossipov D.A., Brännvall K., Forsberg-Nilsson K., Hilborn J. (2007). Formation of the first injectable poly(vinyl alcohol) hydrogel by mixing of functional PVA precursors. J. Appl. Polym. Sci..

[77] Engler A.J., Sen S., Sweeney H.L., Discher D.E. (2006). Matrix Elasticity Directs Stem Cell Lineage Specification. Cell.

[78] Hayward A.S., Sano N., Przyborski S.A., Cameron N.R. (2013). Acrylic-Acid-Functionalized PolyHIPE Scaffolds for Use in 3D Cell Culture. Macromol. Rapid Commun..

[79] Nguyen K.T., West J.L. (2002). Photopolymerizable hydrogels for tissue engineering applications. Biomaterials.

[80] Latxague L., Ramin M.A., Appavoo A., Berto P., Maisani M., Ehret C., Chassande O., Barthélémy P. (2015). Control of Stem-Cell Behavior by Fine Tuning the Supramolecular Assemblies of Low-Molecular-Weight Gelators. Angew. Chem. Int. Ed..

[81] A Versatile Carbohydrate Based Gelator for Oil Water Separation, Nanoparticle Synthesis and Dye Removal—New Journal of Chemistry (RSC Publishing). https://pubs.rsc.org/en/content/articlelanding/2017/NJ/C6NJ03520E.

[82] Rajkamal R., Pathak N.P., Chatterjee D., Paul A., Yadav S. (2016). Arabinose based gelators: Rheological characterization of the gels and phase selective organogelation of crude-oil. RSC Adv..

[83] Chalard A., Vaysse L., Joseph P., Malaquin L., Souleille S., Lonetti B., Sol J.-C., Loubinoux I., Fitremann J. (2018). Simple Synthetic Molecular Hydrogels from Self-Assembling Alkylgalactonamides as Scaffold for 3D Neuronal Cell Growth. ACS Appl. Mater. Interfaces.

[84] Wang W., Wang H., Ren C., Wang J., Tan M., Shen J., Yang Z., Wang P.G., Wang L. (2011). A saccharide-based supramolecular hydrogel for cell culture. Carbohydr. Res..

[85] Ryadnov M. (2007). Peptide α-helices for synthetic nanostructures. Biochem. Soc. Trans..

[86] Colombo G., Soto P., Gazit E. (2007). Peptide self-assembly at the nanoscale: A challenging target for computational and experimental biotechnology. Trends Biotechnol..

[87] Manandhar A., Kang M., Chakraborty K., Tang P.K., LoVerde S.M. (2017). Molecular simulations of peptide amphiphiles. Org. Biomol. Chem..

[88] Jayawarna V., Richardson S.M., Hirst A.R., Hodson N.W., Saiani A., Gough J.E., Ulijn R.V. (2009). Introducing chemical functionality in Fmoc-peptide gels for cell culture. Acta Biomater..

[89] Gao J., Tang C., ElSawy M.A., Smith A.M., Miller A.F., Saiani A. (2017). Controlling Self-Assembling Peptide Hydrogel Properties through Network Topology. Biomacromolecules.

[90] Araki K., Yoshikawa I. (2005). Nucleobase-Containing Gelators. Top. Curr. Chem..

[91] Skilling K.J., Ndungu A., Kellam B., Ashford M., Bradshaw T.D., Marlow M. (2014). Gelation properties of self-assembling N-acyl modified cytidine derivatives. J. Mater. Chem. B.

[92] Alies B., Ouelhazi M.A., Patwa A.N., Verget J., Navailles L., Desvergnes V., Barthelemy P. (2018). Cytidine- and guanosine-based nucleotide–lipids. Org. Biomol. Chem..

[93] Campins N., Dieudonné P., Grinstaff M.W., Barthélémy P. (2007). Nanostructured assemblies from nucleotide-based amphiphiles. New J. Chem..

[94] Patel S., Volpe A.B., Awwad S., Schätzlein A.G., Haider S., Liu B., Uchegbu I.F. (2020). A Self-Assembling Lipidic Peptide and Selective Partial V2 Receptor Agonist Inhibits Urine Production. Sci. Rep..

[95] Zhang S. (2017). Discovery and design of self-assembling peptides. Interface Focus.

[96] Yang Z., Xu H., Zhao X. (2020). Designer Self-Assembling Peptide Hydrogels to Engineer 3D Cell Microenvironments for Cell Constructs Formation and Precise Oncology Remodeling in Ovarian Cancer. Adv. Sci..

[97] Qiu F., Chen Y., Tang C., Zhao X. (2018). Amphiphilic peptides as novel nanomaterials: Design, self-assembly and application. Int. J. Nanomed..

[98] Latxague L., Gaubert A., Maleville D., Baillet J., Ramin M.A., Barthélémy P. (2016). Carbamate-Based Bolaamphiphile as Low-Molecular-Weight Hydrogelators. Gels.

[99] Latxague L., Dalila M.-J., Patwa A., Ziane S., Chassande O., Godeau G., Barthélémy P. (2012). Glycoside nucleoside lipids (GNLs): An intrusion into the glycolipids’ world?. Comptes Rendus Chim..

[100] Nagarajan R. (1987). Self-Assembly of Bola Amphiphiles. Chem. Eng. Commun..

[101] Ochi R., Kurotani K., Ikeda M., Kiyonaka S., Hamachi I. (2013). Supramolecular hydrogels based on bola-amphiphilic glycolipids showing color change in response to glycosidases. Chem. Commun..

[102] Ramakanth I., Patnaik A. (2012). Novel Two-Component Gels of Cetylpyridinium Chloride and the Bola-amphiphile 6-Amino Caproic Acid: Phase Evolution and Mechanism of Gel Formation. J. Phys. Chem. B.

[103] Nebot V.J., Armengol J., Smets J., Prieto S.F., Escuder B., Miravet J.F. (2012). Molecular Hydrogels from Bolaform Amino Acid Derivatives: A Structure-Properties Study Based on the Thermodynamics of Gel Solubilization. Chem. Eur. J..

[104] Ramin M.A., Latxague L., Sindhu K.R., Chassande O., Barthélémy P. (2017). Low molecular weight hydrogels derived from urea based-bolaamphiphiles as new injectable biomaterials. Biomaterials.

[105] Chen X., Fuchs H. (2015). Soft Matter Nanotechnology: From Structure to Function.

[106] Jain N., Goldschmidt V., Oncul S., Arntz Y., Duportail G., Mély Y., Klymchenko A.S. (2012). Lactose-ornithine bolaamphiphiles for efficient gene delivery in vitro. Int. J. Pharm..

[107] Griffin D.R., Weaver W.M., Scumpia P.O., Di Carlo D., Segura T. (2015). Accelerated wound healing by injectable microporous gel scaffolds assembled from annealed building blocks. Nat. Mater..

[108] Ziane S., Schlaubitz S., Miraux S., Patwa A., Lalande C., Bilem I., Lepreux S., Rousseau B., Le Meins J.-F., Latxague L. (2012). A thermosensitive low molecular weight hydrogel as scaffold for tissue engineering. Eur. Cells Mater..

[109] Mirabelli P., Coppola L., Salvatore M. (2019). Cancer Cell Lines Are Useful Model Systems for Medical Research. Cancers.

[110] Shetab-Bou S.V., Abdollahi M. (2012). Current Concerns on the Validity of in vitro Models that use Transformed Neoplastic Cells in Pharmacology and Toxicology. Int. J. Pharmacol..

[111] Liu Z., Zhu L., Thakkar S., Roberts R., Tong W. (2019). Can Transcriptomic Profiles from Cancer Cell Lines Be Used for Toxicity Assessment?. Chem. Res. Toxicol..

[112] Hirschhaeuser F., Menne H., Dittfeld C., West J., Mueller-Klieser W., Kunz-Schughart L.A. (2010). Multicellular tumor spheroids: An underestimated tool is catching up again. J. Biotechnol..

[113] Bonnans C., Chou J., Werb Z. (2014). Remodelling the extracellular matrix in development and disease. Nat. Rev. Mol. Cell Biol..

[114] Hamilton G., Rath B. (2018). Applicability of tumor spheroids for in vitro chemosensitivity assays. Expert Opin. Drug Metab. Toxicol..

[115] Ediriwickrema A., Saltzman W.M. (2015). Nanotherapy for Cancer: Targeting and Multifunctionality in the Future of Cancer Therapies. ACS Biomater. Sci. Eng..

[116] Ng T.S., Garlin M.A., Weissleder R., Miller M.A. (2020). Improving nanotherapy delivery and action through image-guided systems pharmacology. Theranostics.

[117] Schwachöfer J.H. (1990). Multicellular tumor spheroids in radiotherapy research (review). Anticancer Res..

[118] Bahadar H., Maqbool F., Niaz K., Abdollahi M. (2016). Toxicity of Nanoparticles and an Overview of Current Experimental Models. Iran. Biomed. J..

[119] Fröhlich E. (2018). Comparison of conventional and advanced in vitro models in the toxicity testing of nanoparticles. Artif. Cells Nanomed. Biotechnol..

[120] Peng F., Setyawati M.I., Tee J.K., Ding X., Wang J., Nga M.E., Ho H.K., Leong D.T. (2019). Nanoparticles promote in vivo breast cancer cell intravasation and extravasation by inducing endothelial leakiness. Nat. Nanotechnol..

[121] Von Maltzahn G., Park J.-H., Lin K.Y.-M., Singh N., Schwöppe C., Mesters R.M., Berdel W.E., Ruoslahti E., Sailor M.J., Bhatia S.N. (2011). Nanoparticles that communicate in vivo to amplify tumour targeting. Nat. Mater..

[122] Chai Q., Jiao Y., Yu X. (2017). Hydrogels for Biomedical Applications: Their Characteristics and the Mechanisms behind Them. Gels.

[123] Prince E., Kumacheva E. (2019). Design and applications of man-made biomimetic fibrillar hydrogels. Nat. Rev. Mater..

[124] Saleh A., Marhuenda E., Fabre C., Hassani Z., De Weille J., Boukhaddaoui H., Guelfi S., Maldonado I.L., Hugnot J.-P., Duffau H. (2019). A novel 3D nanofibre scaffold conserves the plasticity of glioblastoma stem cell invasion by regulating galectin-3 and integrin-β1 expression. Sci. Rep..

[125] Li Y., Kumacheva E. (2018). Hydrogel microenvironments for cancer spheroid growth and drug screening. Sci. Adv..

[126] Huang B.-W., Gao J.-Q. (2018). Application of 3D cultured multicellular spheroid tumor models in tumor-targeted drug delivery system research. J. Control. Release.

[127] Imamura Y., Mukohara T., Shimono Y., Funakoshi Y., Chayahara N., Toyoda M., Kiyota N., Takao S., Kono S., Nakatsura T. (2015). Comparison of 2D- and 3D-culture models as drug-testing platforms in breast cancer. Oncol. Rep..

[128] Fischbach C., Chen R., Matsumoto T., Schmelzle T., Brugge J.S., Polverini P.J., Mooney D.J. (2007). Engineering tumors with 3D scaffolds. Nat. Methods.

[129] Chaicharoenaudomrung N., Kunhorm P., Noisa P. (2019). Three-dimensional cell culture systems as an in vitro platform for cancer and stem cell modeling. World J. Stem Cells.

[130] Balcão V.M., Barreira S.V.P., Nunes T.M., Chaud M.V., Tubino M., Vila M.M.D.C. (2013). Carbohydrate Hydrogels with Stabilized Phage Particles for Bacterial Biosensing: Bacterium Diffusion Studies. Appl. Biochem. Biotechnol..

[131] Sandrin D., Wagner D., Sitta C.E., Thoma R., Felekyan S., Hermes H.E., Janiak C., Amadeu N.D.S., Kühnemuth R., Löwen H. (2016). Diffusion of macromolecules in a polymer hydrogel: From microscopic to macroscopic scales. Phys. Chem. Chem. Phys..

[132] Engberg K., Frank C.W. (2011). Protein diffusion in photopolymerized poly(ethylene glycol) hydrogel networks. Biomed. Mater..

[133] Zhou Y., Li J., Zhang Y., Dong D., Zhang E., Ji F., Qin Z., Yang J., Yao F. (2017). Establishment of a Physical Model for Solute Diffusion in Hydrogel: Understanding the Diffusion of Proteins in Poly(sulfobetaine methacrylate) Hydrogel. J. Phys. Chem. B.

[134] Golmohamadi M., Wilkinson K.J. (2013). Diffusion of ions in a calcium alginate hydrogel-structure is the primary factor controlling diffusion. Carbohydr. Polym..

[135] Faucher S., Le Coustumer P., Lespes G. (2018). Nanoanalytics: History, concepts, and specificities. Environ. Sci. Pollut. Res..

[136] (2011). 14:00–17:00 ISO/TS 80004-4. https://www.iso.org/cms/render/live/en/sites/isoorg/contents/data/standard/05/21/52195.html.

[137] Bruinink A., Wang J., Wick P. (2015). Effect of particle agglomeration in nanotoxicology. Arch. Toxicol..

[138] Sharma V.K. (2009). Aggregation and toxicity of titanium dioxide nanoparticles in aquatic environment—A Review. J. Environ. Sci. Health Part A.

[139] Baalousha M., Yang Y., Vance M.E., Colman B.P., McNeal S., Xu J., Blaszczak J., Steele M., Bernhardt E., Hochella M.F. (2016). Outdoor urban nanomaterials: The emergence of a new, integrated, and critical field of study. Sci. Total Environ..

[140] Shandilya N., Le Bihan O., Bressot C., Morgeneyer M. (2015). Emission of Titanium Dioxide Nanoparticles from Building Materials to the Environment by Wear and Weather. Environ. Sci. Technol..

[141] Babaizadeh H., Hassan M. (2013). Life cycle assessment of nano-sized titanium dioxide coating on residential windows. Constr. Build. Mater..

[142] Windler L., Lorenz C., Von Goetz N., Hungerbühler K., Amberg M., Heuberger M., Nowack B. (2012). Release of Titanium Dioxide from Textiles during Washing. Environ. Sci. Technol..

[143] Osmond M.J., McCall M.J. (2009). Zinc oxide nanoparticles in modern sunscreens: An analysis of potential exposure and hazard. Nanotoxicology.

[144] Corinaldesi C., Marcellini F., Nepote E., Damiani E., Danovaro R. (2018). Impact of inorganic UV filters contained in sunscreen products on tropical stony corals (*Acropora* spp.). Sci. Total Environ..

[145] Sadik O.A. (2012). Anthropogenic nanoparticles in the environment. Environ. Sci. Process. Impacts.

[146] Harrison R.M., MacKenzie A.R., Xu H., Alam M.S., Nikolova I., Zhong J., Singh A., Zeraati-Rezaei S., Stark C., Beddows D.C.S. (2018). Diesel exhaust nanoparticles and their behaviour in the atmosphere. Proc. R. Soc. A Math. Phys. Eng. Sci..

[147] Kim J.S., Kuk E., Yu K.N., Kim J.-H., Park S.J., Lee H.J., Kim S.H., Park Y.K., Park Y.H., Hwang C.-Y. (2007). Antimicrobial effects of silver nanoparticles. Nanomed. Nanotechnol. Biol. Med..

[148] Becheri A., Dürr M., Nostro P.L., Baglioni P. (2008). Synthesis and characterization of zinc oxide nanoparticles: Application to textiles as UV-absorbers. J. Nanoparticle Res..

[149] Smijs T.G., Pavel S. (2011). Titanium dioxide and zinc oxide nanoparticles in sunscreens: Focus on their safety and effectiveness. Nanotechnol. Sci. Appl..

[150] Egerton T., Christensen P., Kosa S., Onoka B., Harper J., Tinlin J. (2006). Photoelectrocatalysis by titanium dioxide for water treatment. Int. J. Environ. Pollut..

[151] Chertok B., Moffat B.A., David A.E., Yu F., Bergemann C., Ross B.D., Yang V.C. (2008). Iron oxide nanoparticles as a drug delivery vehicle for MRI monitored magnetic targeting of brain tumors. Biomaterials.

[152] Chen J., Poon C.-S. (2009). Photocatalytic activity of titanium dioxide modified concrete materials—Influence of utilizing recycled glass cullets as aggregates. J. Environ. Manag..

[153] Shen S., Burton M., Jobson B., Haselbach L. (2012). Pervious concrete with titanium dioxide as a photocatalyst compound for a greener urban road environment. Constr. Build. Mater..

[154] You H., Yang S., Ding B., Yang H. (2013). Synthesis of colloidal metal and metal alloy nanoparticles for electrochemical energy applications. Chem. Soc. Rev..

[155] Frey N.A., Peng S., Cheng K., Sun S. (2009). Magnetic nanoparticles: Synthesis, functionalization, and applications in bioimaging and magnetic energy storage. Chem. Soc. Rev..

[156] Webb J.A., Bardhan R. (2014). Emerging advances in nanomedicine with engineered gold nanostructures. Nanoscale.

[157] Das S., Dowding J.M., Klump K.E., McGinnis J.F., Self W., Seal S. (2013). Cerium oxide nanoparticles: Applications and prospects in nanomedicine. Nanomedicine.

[158] Guarino-Hotz M., Zhang J.Z. (2021). Structural control and biomedical applications of plasmonic hollow gold nanospheres: A mini review. Wiley Interdiscip. Rev. Nanomed. Nanobiotechnol..

[159] Ramazanov M., Karimova A., Shirinova H. (2020). Magnetism for Drug Delivery, MRI and Hyperthermia Applications: A Review. Biointerface Res. Appl. Chem..

[160] Cormode D.P., Naha P.C., Fayad Z.A. (2014). Nanoparticle contrast agents for computed tomography: A focus on micelles. Contrast Media Mol. Imaging.

[161] Hochella M.F., Mogk D.W., Ranville J., Allen I.C., Luther G.W., Marr L.C., McGrail B.P., Murayama M., Qafoku N.P., Rosso K.M. (2019). Natural, incidental, and engineered nanomaterials and their impacts on the Earth system. Science.

[162] Rahman Q., Lohani M., Dopp E., Pemsel H., Jonas L., Weiss D.G., Schiffmann D. (2002). Evidence that ultrafine titanium dioxide induces micronuclei and apoptosis in Syrian hamster embryo fibroblasts. Environ. Health Perspect..

[163] Yamamoto A., Honma R., Sumita M., Hanawa T. (2003). Cytotoxicity evaluation of ceramic particles of different sizes and shapes. J. Biomed. Mater. Res..

[164] Pan Y., Neuss S., Leifert A., Fischler M., Wen F., Simon U., Schmid G., Brandau W., Jahnen-Dechent W. (2007). Size-Dependent Cytotoxicity of Gold Nanoparticles. Small.

[165] Vevers W.F., Jha A.N. (2008). Genotoxic and cytotoxic potential of titanium dioxide (TiO_2_) nanoparticles on fish cells in vitro. Ecotoxicology.

[166] Asharani P.V., Mun G.L.K., Hande M.P., Valiyaveettil S. (2008). Cytotoxicity and Genotoxicity of Silver Nanoparticles in Human Cells. ACS Nano.

[167] Foldbjerg R.B., Dang D.A., Autrup H. (2010). Cytotoxicity and genotoxicity of silver nanoparticles in the human lung cancer cell line, A549. Arch. Toxicol..

[168] Park M.V., Neigh A.M., Vermeulen J.P., De La Fonteyne L.J., Verharen H.W., Briedé J.J., Van Loveren H., De Jong W.H. (2011). The effect of particle size on the cytotoxicity, inflammation, developmental toxicity and genotoxicity of silver nanoparticles. Biomaterials.

[169] Helmlinger J., Sengstock C., Groß-Heitfeld C., Mayer C., Schildhauer T.A., Köller M., Epple M. (2016). Silver nanoparticles with different size and shape: Equal cytotoxicity, but different antibacterial effects. RSC Adv..

[170] Akhtar M.J., Ahamed M., Alhadlaq H.A., Alrokayan S.A. (2018). MgO nanoparticles cytotoxicity caused primarily by GSH depletion in human lung epithelial cells. J. Trace Elem. Med. Biol..

[171] Lovern S.B., Klaper R. (2006). Daphnia Magna Mortality When Exposed to Titanium Dioxide and Fullerene (C60) Nanoparticles. Environ. Toxicol. Chem..

[172] Wang J., Zhou G., Tiancheng W., Yu H., Wang T., Ma Y., Jiangxue W., Gao Y., Li Y., Sun J. (2007). Acute toxicity and biodistribution of different sized titanium dioxide particles in mice after oral administration. Toxicol. Lett..

[173] Chen T.-H., Lin C.-Y., Tseng M.-C. (2011). Behavioral effects of titanium dioxide nanoparticles on larval zebrafish (*Danio rerio*). Mar. Pollut. Bull..

[174] Tiwari D.K., Jin T., Behari J. (2010). Dose-dependent in-vivo toxicity assessment of silver nanoparticle in Wistar rats. Toxicol. Mech. Methods.

[175] Mahmoudi M., Hofmann H., Rothen-Rutishauser B., Petri-Fink A. (2011). Assessing the In Vitro and In Vivo Toxicity of Superparamagnetic Iron Oxide Nanoparticles. Chem. Rev..

[176] Simpson C.A., Salleng K.J., Cliffel D.E., Feldheim D.L. (2013). In vivo toxicity, biodistribution, and clearance of glutathione-coated gold nanoparticles. Nanomed. Nanotechnol. Biol. Med..

[177] Askri D., Ouni S., Galai S., Chovelon B., Arnaud J., Lehmann S.G., Sakly M., Sève M., Amara S. (2018). Sub-acute intravenous exposure to Fe_2_O_3_ nanoparticles does not alter cognitive performances and catecholamine levels, but slightly disrupts plasma iron level and brain iron content in rats. J. Trace Elem. Med. Biol..

[178] Hussain S., Hess K., Gearhart J., Geiss K., Schlager J. (2005). In vitro toxicity of nanoparticles in BRL 3A rat liver cells. Toxicol. In Vitro.

[179] Teodoro J.S., Simões A.M., Duarte F., Rolo A.P., Murdoch R.C., Hussain S.M., Palmeira C.M. (2011). Assessment of the toxicity of silver nanoparticles in vitro: A mitochondrial perspective. Toxicol. In Vitro.

[180] Avalos A., Haza A.I., Mateo D., Morales P. (2013). Cytotoxicity and ROS production of manufactured silver nanoparticles of different sizes in hepatoma and leukemia cells. J. Appl. Toxicol..

[181] Kaba S.I., Egorova E.M. (2015). In vitro studies of the toxic effects of silver nanoparticles on HeLa and U937 cells. Nanotechnol. Sci. Appl..

[182] Reeves J.F., Davies S.J., Dodd N.J., Jha A.N. (2008). Hydroxyl radicals (OH) are associated with titanium dioxide (TiO_2_) nanoparticle-induced cytotoxicity and oxidative DNA damage in fish cells. Mutat. Res. Mol. Mech. Mutagen..

[183] Osman I.F., Baumgartner A., Cemeli-Carratala E., Fletcher J.N., Anderson D. (2010). Genotoxicity and cytotoxicity of zinc oxide and titanium dioxide in HEp-2 cells. Nanomedicine.

[184] Thurn K.T., Arora H., Paunesku T., Wu A., Brown E.M., Doty C., Kremer J., Woloschak G. (2011). Endocytosis of titanium dioxide nanoparticles in prostate cancer PC-3M cells. Nanomed. Nanotechnol. Biol. Med..

[185] Chen L., Zhou L., Liu Y., Deng S., Wu H., Wang G. (2012). Toxicological effects of nanometer titanium dioxide (nano-TiO_2_) on *Chlamydomonas reinhardtii*. Ecotoxicol. Environ. Saf..

[186] Srivastava R.K., Rahman Q., Kashyap M.P., Singh A.K., Jain G., Jahan S., Lohani M., Lantow M., Pant A.B. (2012). Nano-titanium dioxide induces genotoxicity and apoptosis in human lung cancer cell line, A549. Hum. Exp. Toxicol..

[187] El-Said K.S., Ali E.M., Kanehira K., Taniguchi A. (2014). Molecular mechanism of DNA damage induced by titanium dioxide nanoparticles in toll-like receptor 3 or 4 expressing human hepatocarcinoma cell lines. J. Nanobiotechnol..

[188] Wang Y., Cui H., Zhou J., Li F., Wang J., Chen M., Liu Q. (2014). Cytotoxicity, DNA damage, and apoptosis induced by titanium dioxide nanoparticles in human non-small cell lung cancer A549 cells. Environ. Sci. Pollut. Res..

[189] Biondi M., Guarnieri D., Yu H., Belli V., Netti P.A. (2013). Sub-100 nm biodegradable nanoparticles: In vitro release features and toxicity testing in 2D and 3D cell cultures. Nanotechnology.

[190] Lopes V.R., Loitto V., Audinot J.-N., Bayat N., Gutleb A.C., Cristobal S. (2016). Dose-dependent autophagic effect of titanium dioxide nanoparticles in human HaCaT cells at non-cytotoxic levels. J. Nanobiotechnol..

[191] Sun Q., Tan D., Ze Y., Sang X., Liu X., Gui S., Cheng Z., Cheng J., Hu R., Gao G. (2012). Pulmotoxicological effects caused by long-term titanium dioxide nanoparticles exposure in mice. J. Hazard. Mater..

[192] Zhao L., Zhu Y., Chen Z., Xu H., Zhou J., Tang S., Xu Z., Kong F., Li X., Zhang Y. (2018). Cardiopulmonary effects induced by occupational exposure to titanium dioxide nanoparticles. Nanotoxicology.

[193] Qu Y., Lü X. (2009). Aqueous synthesis of gold nanoparticles and their cytotoxicity in human dermal fibroblasts–fetal. Biomed. Mater..

[194] Connor E.E., Mwamuka J., Gole A., Murphy C.J., Wyatt M.D. (2005). Gold Nanoparticles Are Taken Up by Human Cells but Do Not Cause Acute Cytotoxicity. Small.

[195] Escudero-Francos M.A., Cepas V., González-Menédez P., Badía-Laíño R., Díaz-García M.E., Sainz R.M., Mayo J.C., Hevia D. (2017). Cellular Uptake and Tissue Biodistribution of Functionalized Gold Nanoparticles and Nanoclusters. J. Biomed. Nanotechnol..

[196] Susewind J., Carvalho-Wodarz C.D.S., Repnik U., Collnot E.-M., Schneider-Daum N., Griffiths G.W., Lehr C.-M. (2015). A 3D co-culture of three human cell lines to model the inflamed intestinal mucosa for safety testing of nanomaterials. Nanotoxicology.

[197] Theumer A., Grafe C., Bähring F., Bergemann C., Hochhaus A., Clement J.H. (2015). Superparamagnetic iron oxide nanoparticles exert different cytotoxic effects on cells grown in monolayer cell culture versus as multicellular spheroids. J. Magn. Magn. Mater..

[198] Le V.-M., Lang M.-D., Shi W.-B., Liu J.-W. (2014). A collagen-based multicellular tumor spheroid model for evaluation of the efficiency of nanoparticle drug delivery. Artif. Cells Nanomed. Biotechnol..

[199] Wu Z., Guan R., Tao M., Lyu F., Cao G., Liu M., Gao J. (2017). Assessment of the toxicity and inflammatory effects of different-sized zinc oxide nanoparticles in 2D and 3D cell cultures. RSC Adv..

[200] Chen B., Wang J., Chen Y., Ding J., Xia G., Gao C., Cheng J., Jin N., Zhou Y., Li X. (2010). Pharmacokinetic parameters and tissue distribution of magnetic Fe_3_O_4_ nanoparticles in mice. Int. J. Nanomed..

[201] Coccini T., Caloni F., Cando L.J.R., De Simone U. (2016). Cytotoxicity and proliferative capacity impairment induced on human brain cell cultures after short- and long-term exposure to magnetite nanoparticles. J. Appl. Toxicol..

[202] Sambale F., Lavrentieva A., Stahl F., Blume C., Stiesch M., Kasper C., Bahnemann D., Scheper T. (2015). Three dimensional spheroid cell culture for nanoparticle safety testing. J. Biotechnol..

[203] Yu M., Huang S., Yu K.J., Clyne A.M. (2012). Dextran and Polymer Polyethylene Glycol (PEG) Coating Reduce Both 5 and 30 nm Iron Oxide Nanoparticle Cytotoxicity in 2D and 3D Cell Culture. Int. J. Mol. Sci..

[204] Chia S.L., Tay C.Y., Setyawati M.I., Leong D.T. (2014). Biomimicry 3D Gastrointestinal Spheroid Platform for the Assessment of Toxicity and Inflammatory Effects of Zinc Oxide Nanoparticles. Small.

[205] De Simone U., Roccio M., Gribaldo L., Spinillo A., Caloni F., Coccini T. (2018). Human 3D Cultures as Models for Evaluating Magnetic Nanoparticle CNS Cytotoxicity after Short- and Repeated Long-Term Exposure. Int. J. Mol. Sci..

[206] Lee J., Lilly G.D., Doty R.C., Podsiadlo P., Kotov N.A. (2009). In vitro Toxicity Testing of Nanoparticles in 3D Cell Culture. Small.

[207] Weiswald L.-B., Bellet D., Dangles-Marie V. (2015). Spherical Cancer Models in Tumor Biology. Neoplasia.

[208] He H., Liu C., Liu Y., Liu X., Wu Y., Fan J., Zhao L., Cao Y. (2019). Mathematical modeling of the heterogeneous distributions of nanomedicines in solid tumors. Eur. J. Pharm. Biopharm..

[209] Guimarães C.F., Gasperini L., Marques A.P., Reis R.L. (2020). The stiffness of living tissues and its implications for tissue engineering. Nat. Rev. Mater..

[210] Lu H., Stenzel M.H. (2018). Multicellular Tumor Spheroids (MCTS) as a 3D In Vitro Evaluation Tool of Nanoparticles. Small.

[211] Cao Y., Gong Y., Liu L., Zhou Y., Fang X., Zhang C., Li Y., Li J. (2017). The use of human umbilical vein endothelial cells (HUVECs) as an in vitro model to assess the toxicity of nanoparticles to endothelium: A review. J. Appl. Toxicol..

[212] Sindhwani S., Syed A.M., Ngai J., Kingston B.R., Maiorino L., Rothschild J., Macmillan P., Zhang Y., Rajesh N.U., Hoang T. (2020). The entry of nanoparticles into solid tumours. Nat. Mater..

[213] Gollwitzer C., Bartczak D., Goenaga-Infante H., Kestens V., Krumrey M., Minelli C., Pálmai M., Ramaye Y., Roebben G., Sikora A. (2016). A comparison of techniques for size measurement of nanoparticles in cell culture medium. Anal. Methods.

[214] Chen Z.P., Xu R.Z., Zhang Y., Gu N. (2008). Effects of Proteins from Culture Medium on Surface Property of Silanes-Functionalized Magnetic Nanoparticles. Nanoscale Res. Lett..

[215] Kato H., Fujita K., Horie M., Suzuki M., Nakamura A., Endoh S., Yoshida Y., Iwahashi H., Takahashi K., Kinugasa S. (2010). Dispersion characteristics of various metal oxide secondary nanoparticles in culture medium for in vitro toxicology assessment. Toxicol. In Vitro.

[216] Dubiak-Szepietowska M., Karczmarczyk A., Jönsson-Niedziółka M., Winckler T., Feller K.-H. (2016). Development of complex-shaped liver multicellular spheroids as a human-based model for nanoparticle toxicity assessment in vitro. Toxicol. Appl. Pharmacol..

[217] Chauhan S., Manivasagam G., Kumar P., Ambasta R.K. (2019). Cellular Toxicity of Mesoporous Silica Nanoparticle in SHSY5Y and BMMNCs Cell. Pharm. Nanotechnol..

[218] Braun K., Stürzel C.M., Biskupek J., Kaiser U., Kirchhoff F., Lindén M. (2018). Comparison of different cytotoxicity assays for in vitro evaluation of mesoporous silica nanoparticles. Toxicol. In Vitro.

[219] Bonnier F., Keating M., Wróbel T., Majzner K., Baranska M., Garcia-Munoz A., Blanco A., Byrne H.J. (2015). Cell viability assessment using the Alamar blue assay: A comparison of 2D and 3D cell culture models. Toxicol. In Vitro.

[220] Eustaquio T., Leary J.F. (2012). Single-Cell Nanotoxicity Assays of Superparamagnetic Iron Oxide Nanoparticles. Adv. Struct. Saf. Stud..

[221] Sonmez E., Cacciatore I., Bakan F., Turkez H., I Mohtar Y., Togar B., Stefano A.D. (2016). Toxicity assessment of hydroxyapatite nanoparticles in rat liver cell model in vitro. Hum. Exp. Toxicol..

[222] Mota A., Hemati-Dinarvand M., Taheraghdam A.A., Nejabati H.R., Ahmadi R., Ghasemnejad T., Hasanpour M., Valilo M. (2019). Association of Paraoxonse1 (PON1) Genotypes with the Activity of PON1 in Patients with Parkinson’s Disease. Acta Neurol. Taiwanica.

